# Nrf2 activator peptide protects the brain from cerebral vascular dysfunction in alcohol ingestion

**DOI:** 10.1172/jci.insight.188004

**Published:** 2026-02-17

**Authors:** Bibhuti Ballav Saikia, Saleena Alikunju, Yemin A. Poovanthodi, Zayan Kassim, P.M. Abdul Muneer

**Affiliations:** 1 JFK Neuroscience Institute, Hackensack Meridian Health JFK University Medical Center, Edison, New Jersey, USA.; 2 Miami Project to Cure Paralysis, Lois Pope LIFE Center, Miller School of Medicine, University of Miami, Miami, Florida, USA.; 3 Laboratory of CNS Injury and Molecular Therapy, Department of Biomedical Engineering, Florida International University, Miami, Florida, USA.

**Keywords:** Neuroscience, Vascular biology, Cell migration/adhesion, Cellular immune response, Therapeutics

## Abstract

Oxidative signaling is a central mechanism in alcohol-induced injury and has strong implications for blood-brain barrier (BBB) dysregulation and neuroinflammation. Here, by targeting oxidative signaling, we hypothesized an innovative approach to develop a clinically relevant therapeutic strategy for alleviating alcohol-mediated neurovascular damage. To accomplish this, we enhanced the endogenous activity of nuclear factor E2–related factor 2 (Nrf2) by treatment with a Nrf2 activator III TAT peptide (Nrf2 peptide [NP]) and investigated the neuroprotective role of Nrf2 in promoting antioxidant defense properties and reducing BBB damage and transmigration of leukocytes to the brain following alcohol ingestion. We administered the NP subcutaneously to alcohol-ingested mice and evaluated its therapeutic potential in alleviating alcohol-associated neurovascular impairments. We compared the results with those seen in animals treated with control peptide (random sequence with TAT). The studies showed that the NP treatment preserved the oxidant-antioxidant balance, downregulated ICAM-1 and its receptors, and mitigated BBB damage and leukocyte infiltration into the brain. We validated the effect of the NP in Nrf2-knockout (*Nrf2^−/−^*) mice. Thus, this study demonstrates that NP exerts neurovascular protective effects by regulating the oxidant-antioxidant balance, reducing oxidative stress–induced BBB disruption, and limiting transmigration of immune cells to the brain in a mouse model of alcohol ingestion.

## Abstract 2

In this study, we developed a therapeutic strategy to reduce alcohol-induced neurovascular damage by enhancing Nrf2 activity with an activator peptide, showing its role in boosting antioxidant defenses and reducing blood-brain barrier damage and leukocyte migration.

## Graphical abstract

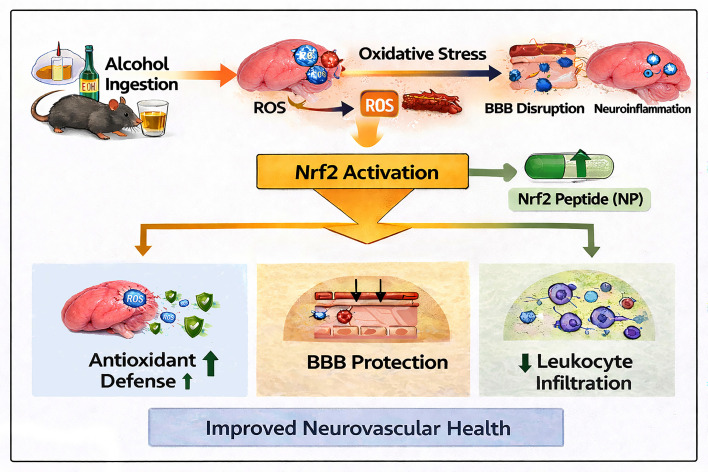

## Introduction

In the central nervous system (CNS), regulation of blood-brain barrier (BBB) integrity is an essential and critical function that prevents the nonspecific infiltration of molecules and cells into the brain (1). BBB damage causes alteration in tight junction proteins and paves the way for the transmigration of immune cells (e.g., leukocytes) across the BBB in various pathological conditions that include HIV-associated dementia (2) and encephalitis (3), multiple sclerosis (4), bacterial meningitis (5), stroke (6), and brain trauma (7, 8). Among the BBB structural components, tight junction proteins such as claudin-5, occludin, and zonula occludens-1 (ZO-1) play crucial roles in maintaining endothelial barrier integrity (1). Oxidative or inflammatory stimuli, such as alcohol exposure, can disrupt these proteins through the activation of matrix metalloproteinases (MMP-2 and MMP-9), leading to increased paracellular permeability (9, 10). In addition, MMP-mediated degradation of vascular endothelial growth factor receptor-2 (VEGFR-2) — a key regulator of angiogenesis and endothelial cell survival — further impairs BBB repair and vascular stability (9). Therefore, understanding how oxidative signaling affects these focal proteins provides important mechanistic insight into alcohol-induced BBB disruption. Oxidative signaling plays a key role in alcohol-induced injury and has strong implications for BBB dysregulation and neuroinflammation (9, 11–14). We have shown that alcohol-mediated oxidative stress plays a major role in BBB dysfunction (11, 13). Oxidative stress activates MMPs that cause disruption of the BBB and neuroinflammation in a rat model of alcohol ingestion (9). Here, activation of MMPs by ethanol (EtOH) leads to degradation of VEGFR-2 and BBB tight junction proteins, promoting impairment of angiogenesis and BBB damage (9). Previous reports from the Haorah laboratory in human brain microvascular endothelial cells and animal models of alcohol use disorder suggest that alcohol activates oxidative stress by enhancing the generation of reactive oxygen species (ROS) and causing BBB damage, which is associated with increased BBB permeability and leukocyte accumulation and infiltration into the brain (9, 11). Therefore, in this study, we hypothesize a clinically relevant therapeutic strategy for alleviating alcohol-mediated neurovascular damage by targeting the oxidative signaling pathway. To accomplish this, we enhanced the endogenous activity of nuclear factor E2–related factor 2 (Nrf2) using Nrf2 activator III TAT peptide (abbreviated as Nrf2 peptide [NP]) and investigated the neuroprotective role of Nrf2 in promoting antioxidant scavenging properties and reducing BBB damage and transmigration of leukocytes into the brain following alcohol ingestion.

Nrf2 is a versatile transcription factor that belongs to the Cap’n’collar family of proteins (15, 16). It plays a central role in modulating the response to various types of stress as it activates the transcription of several genes coding for cellular antioxidants (17), detoxification enzymes (18), drug efflux pumps (19), and other cytoprotective proteins (20). Under normal conditions, Nrf2 is bound to Kelch-like ECH-associated protein-1 (Keap1) in the cytoplasm, leading to its ubiquitination and proteasomal degradation via the Cul3-Rbk1 complex. Under oxidative stress, modification of Keap1 cysteine residues disrupts this interaction, allowing newly synthesized Nrf2 to phosphorylate, translocate into the nucleus, and activate antioxidant response element–dependent (ARE-dependent) genes involved in antioxidant defense and detoxification (21, 22). Remediation of oxidative radicals via Nrf2 induction has already been proposed as a therapeutic strategy for various pathophysiological conditions, including neurodegenerative diseases, cardiovascular disorders, metabolic dysfunctions, and inflammatory conditions (23–25). The protective role of Nrf2 in liver and lung diseases with alcohol abuse has been recently established, with Nrf2 knockdown exacerbating the effects of alcohol on liver injury and necroptosis (26). Similarly, Nrf2 activation through Keap1 knockdown reduces serum triglyceride and hepatic free fatty acid in the livers of ethanol-treated mice (27). In lung tissue, alcohol induces oxidative stress by inhibiting the Nrf2/ARE signaling pathway (28). Recently, we have shown that alcohol reduces the expression level of antioxidant genes HO-1, GPx1, and GSTm1 (29). These antioxidant enzymes are direct transcriptional targets of Nrf2 and are essential for maintaining redox homeostasis in vascular endothelial cells (29). Their loss facilitates oxidative injury and degradation of junctional proteins such as claudin-5 and occludin, thereby contributing to BBB breakdown (30). Although the protective role of Nrf2 against alcohol abuse in liver and lung tissues is well established, there is limited information on the neuroprotective role of Nrf2 in alcohol-induced brain damage.

**Figure 1:**
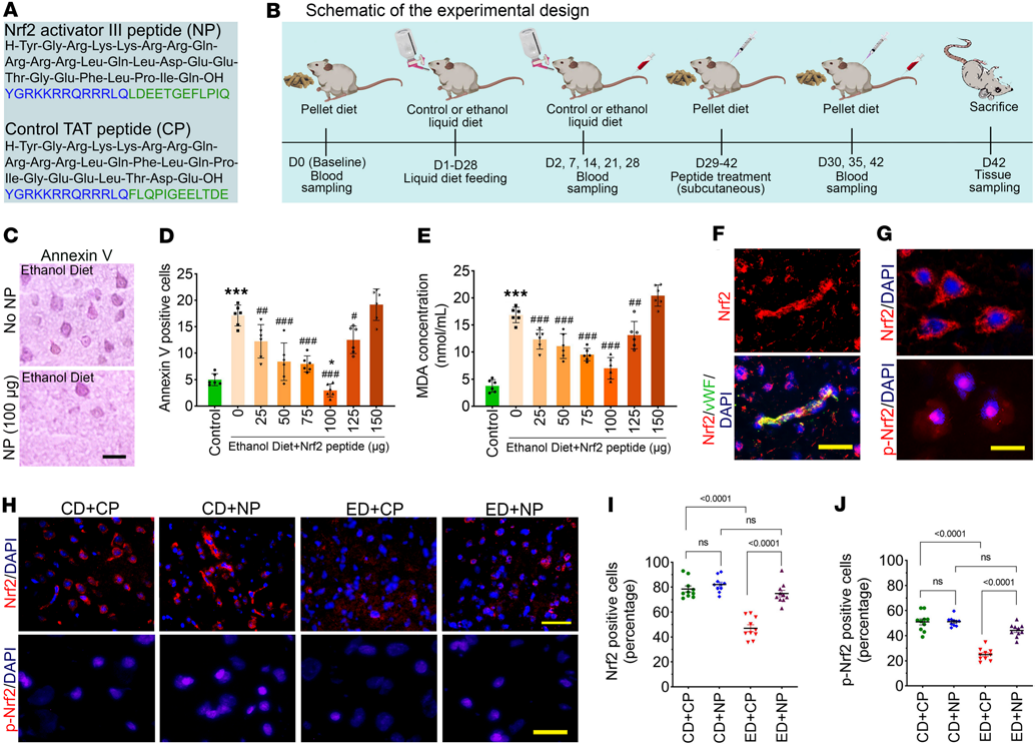
Alcohol attenuates Nrf2 activation. (**A**) Peptide sequences: Nrf2 activator III TAT peptide (NP) and control TAT peptide (CP). (**B**) Schematic representation of the whole experimental procedure. (**C**–**E**) Dose-response test for Nrf2 peptide (NP; 0–150 μg; 1 injection daily) was conducted in mice subjected to a 4-week ethanol diet (ED) regimen; cell death was analyzed by immunostaining with annexin V (**C** and **D**) and ROS-associated lipid peroxidation was analyzed by measurement of malondialdehyde (MDA) levels (**E**) in the brain cortex tissue. (**C**) Annexin V staining (HRP-immunohistochemistry with purple substrate, SK-4600, Vector Laboratories) with or without NP treatment (100 μg). Scale bar: 10 μm. (**D**) Annexin V–positive cells (percentage) in different doses of NP (0–150 μg) (*n* = 6 per group). (**F**) Expression of Nrf2 (red) colocalized with vWF (green) and DAPI in intact brain microvessels of a control diet (CD) animal by immunofluorescence staining (blue). Scale bar: 10 μm. (**G**) Immunostaining of Nrf2 and p-Nrf2 colocalized with DAPI (nucleus) in brain cortex tissue sections that shows the localization of Nrf2 (cytoplasmic) and p-Nrf2 (nuclear). Scale bar: 10 μm. (**H**) Immunofluorescence of Nrf2 (red, top panels) and p-Nrf2 (red, bottom panels) staining merged with DAPI (blue) in CD and ED treated with CP or NP. Scale bars: 50 μm in top panels, 10 μm in bottom panels. (**I** and **J**) Quantification of Nrf2 and p-Nrf2 staining analyzed using ImageJ software (*n* = 10 per group). All values are expressed as mean ± SD. Statistical analysis was performed by 2-way ANOVA followed by Bonferroni’s post hoc test. Statistically significant, *P < 0.05, ***P < 0.001 compared with control (no EtOH or NP); ^#^*P* < 0.05, ^##^*P* < 0.01, ^###^*P* < 0.001 compared with 0 concentration of NP (second bar) in **D** and **E**. In **I** and **J**, exact *P* values are shown between the compared groups.

Peptide-based therapeutic strategies have been pursued in various neuropathologies and disorders, including alcohol abuse and CNS injury (31, 32). Activation of endogenous antioxidant genes that normally combat oxidative damage using peptide activators is a targeted and promising treatment strategy (32, 33). Compared with chemical antioxidants, peptide treatments have fewer side effects and show high therapeutic efficacy in clinical trials (34, 35). Thus, in this study, we propose to improve Nrf2 activity using a synthetic NP (EMD Millipore) (36). Using the same peptide, Steel et al. recently demonstrated enhanced expression levels of antioxidant genes through sustained stabilization and increased nuclear translocation of Nrf2 in THP-1 cells (36). NP is a 14-mer peptide conjugated to a cell-penetrating trans-activating transcriptional activator (TAT) sequence at the C-terminus (complete sequence in Figure 1A) that targets the Nrf2 binding site on Keap1. The peptide with a binding affinity of *K_D_* = 61.9 ± 16.5 nM competitively disrupts Nrf2-Keap1 interaction and stabilizes cytosolic Nrf2 and promotes its nuclear translocation and interaction with ARE (37). TAT sequence facilitates the entry of the peptide into cells, and TAT-fusion proteins or peptides have been shown to retain their biological activity (38), increase the half-life of the peptide, and be able to rapidly access the intracellular space of cultured cells and intact tissues following extracellular applications in vitro and in vivo (38). This NP can cross cell membranes without causing lethal membrane damage (39) and can cross the BBB (40–42). Thus, in this study, besides showing the regulation of the oxidant-antioxidant balance mechanism, we investigated the neurovascular protective actions of NP in reducing oxidative stress–induced pericyte loss, BBB disruption, and transmigration of immune cells into the brain in a mouse model of alcohol ingestion.

## Results

### Determination of optimal dose and evaluation of drug safety.

The peptide sequences used in this study, including the Nrf2 peptide (NP) and the corresponding control peptide (CP), are shown in Figure 1A, and a schematic representation of the experimental design is provided in Figure 1B. Next, we assessed the dose-dependent effects of NP on alcohol-induced endothelial and microvascular cell damage using annexin V staining as a marker of vascular apoptosis. To identify the optimal NP concentration, mice subjected to a 4-week alcohol regimen received daily NP injections (0–150 μg) for 2 consecutive weeks after alcohol exposure. Brain sections were immunostained for annexin V, and annexin V–positive cells were quantified across treatment and control groups. Annexin V–positive cells were markedly reduced at the NP dose of 100 μg per mouse (~4 mg/kg in 100 μL) compared with other doses, indicating decreased apoptotic activity (*P* < 0.0001; Figure 1, C and D). In parallel, ROS-associated lipid peroxidation was assessed by measurement of malondialdehyde (MDA) levels in the brains of NP-treated animals. Among the tested doses, the 100 μg NP treatment produced the most pronounced neuroprotective effect, showing a significant reduction in MDA levels compared with other doses (*P* < 0.0001; Figure 1E). This dose was well tolerated, as indicated by stable body weight, normal grooming and activity, and unaltered serum biochemical markers of oxidative stress and cell death. Together, these results demonstrate both the safety and efficacy of NP at the selected in vivo concentration.

### Alcohol impairs the expression of Nrf2 while Nrf2 peptide treatments activate it.

To test our hypothesis that activation of Nrf2 through NP treatment would suppress BBB dysfunction, vascular permeability, and leukocyte transmigration into the brain following alcohol ingestion, we treated mice daily with a single subcutaneous dose of the NP (100 μg/100 μL). First, activation of Nrf2 by the NP was analyzed using immunofluorescence and Western blotting. We specifically analyzed the expression of Nrf2 in the brain microvessels (Figure 1F). Immunostaining of brain tissue sections showed Nrf2 predominantly in the cytoplasm, while phosphorylated Nrf2 (p-Nrf2) was localized mainly in the nucleus, indicating activation and nuclear translocation (Figure 1G). Next, we analyzed the expression level of Nrf2 and p-Nrf2 in the frontal brain tissue section following alcohol ingestion using immunofluorescence. A 2-way ANOVA revealed significant main effects of diet and treatment on Nrf2 (*F*_(3,36)_ = 28.10, *P* < 0.0001) and p-Nrf2 (*F*_(3,36)_ = 27.72, *P* < 0.0001). Post hoc comparisons indicated that both Nrf2 and p-Nrf2 levels were significantly reduced in ethanol diet–fed (ED-fed) mice compared with the control diet (CD) mice (*P* < 0.0001; Figure 1, H–J). Importantly, NP treatment activated Nrf2 signaling, leading to increased Nrf2 and p-Nrf2 expression in ED+NP mice compared with ED+CP mice (*P* < 0.0001; Figure 1, H–J).

**Figure 2:**
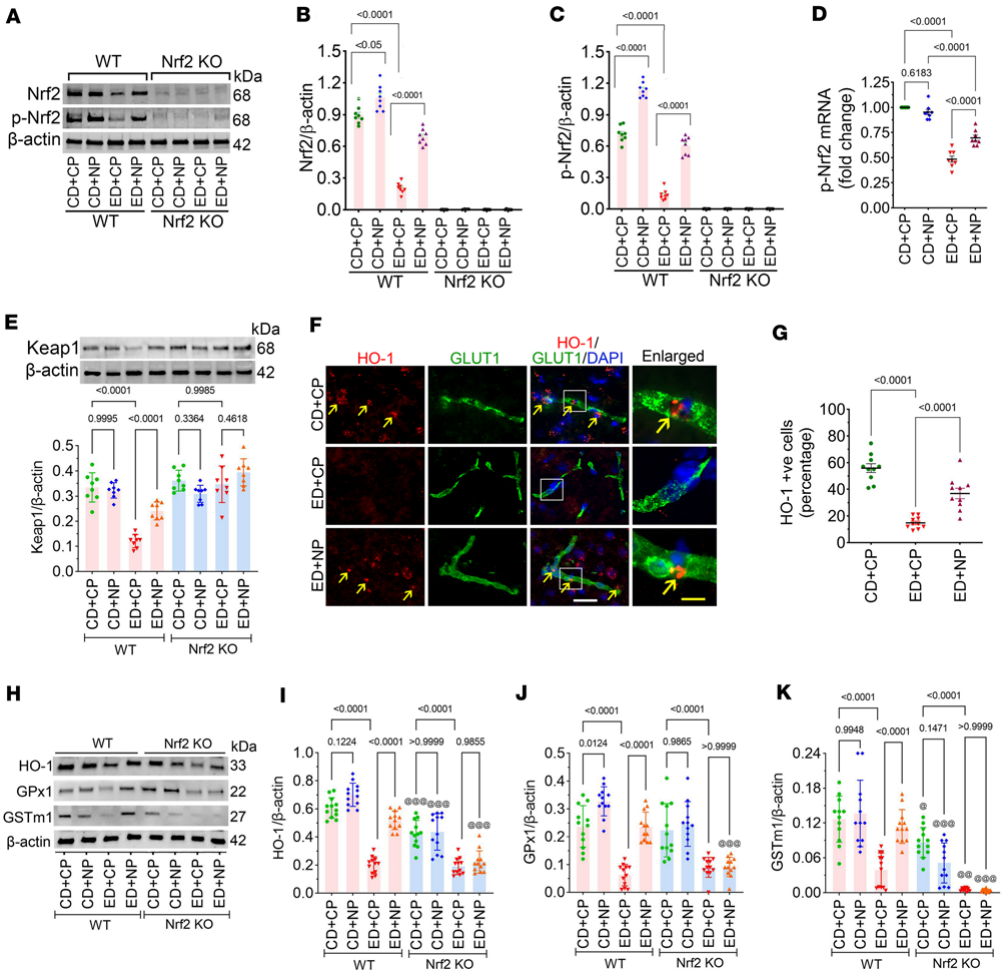
Nrf2 peptide improves expression of antioxidant proteins in alcohol ingestion. (**A**–**C**) Western blot analysis of Nrf2, p-Nrf2, and β-actin from mouse cortex tissue lysate of CD- and ED-fed animals in WT and Nrf2-knockout animals (*Nrf2^–/–^*) with CP or NP treatment. Bar graphs (**B** and **C**) with dot plot show ratio densitometry of Nrf2 and p-Nrf2 to β-actin (*n* = 8 per group). (**D**) mRNA expression level of p-Nrf2 using qPCR from the cortex of CD-fed animals with CP or NP and ED-fed animals with CP or NP (*n* = 8 per group). (**E**) Western blot analysis of Keap1 and β-actin from mouse cortex tissue lysate of CD- and ED-fed animals in WT and Nrf2-knockout animals with CP or NP treatment. Bar graph with dot plot shows ratio densitometry of Keap1 to β-actin (*n* = 8 per group). (**F** and **G**) Immunofluorescence staining of the antioxidant protein HO-1 colocalized with GLUT1 (microvessel marker) and DAPI (nucleus) in intact brain microvessels of mice fed with CD or ED with CP or NP treatments. Yellow arrows show expression of HO-1. Scale bars: white, 50 μm; yellow, 10 μm. (**G**) Quantification of HO-1 staining was analyzed using ImageJ software (*n* = 10 per group). (**H**–**K**) Western blot analysis of antioxidant proteins HO-1, GPx1, and GSTm1 and β-actin from mouse frontal cortex tissue lysate of CD- and ED-fed animals in WT and Nrf2-knockout animals with CP or NP treatment. Bar graphs with dot plot show ratio densitometry of HO-1 (**I**), GPx1 (**J**), and GSTm1 (**K**) to β-actin (*n* = 12 per group). All values are expressed as mean ± SD. Statistical analysis was performed by ANOVA (2-way for **D** and **G**, 3-way for **B**, **C**, **E**, **I**, and **J**) followed by Bonferroni’s post hoc test. *P* < 0.05 was considered statistically significant. ^@^*P* < 0.05, ^@@^*P* < 0.01, ^@@^*P* < 0.001 vs. representative groups in WT animals. Exact *P* values are shown between the compared groups.

Further, Western blot analysis confirmed these findings, showing significantly reduced Nrf2 and p-Nrf2 expression in ED+CP WT group compared with CD+CP WT group animals (*P* < 0.0001; Figure 2, A–C). Notably, NP treatment caused an increased expression of both Nrf2 and p-Nrf2 in the ED+NP WT group compared with the ED+CP group (~3.5-fold for both Nrf2 and p-Nrf2, *P* < 0.0001). Here, we used *Nrf2^−/−^* mice to validate the Nrf2 expression and activation of Nrf2 by NP treatment. Consistent with the genotype, neither Nrf2 nor p-Nrf2 was detected in *Nrf2^−/−^* mice (Figure 2, A–C); however, a very faint signal in *Nrf2^–/–^* samples likely reflects nonspecific binding or residual nonfunctional protein, consistent with basal background levels.

Similarly, the expression level of Nrf2 mRNA by RT-qPCR was reduced to half in ED+CP animals compared with CD+CP control animals (~2.0-fold, *F*_(3,28)_ = 99.07, *P* < 0.0001). NP treatment significantly increased the expression of Nrf2 mRNA in the ED+NP group compared with ED+CP as we observed in the protein expression (~1.5-fold, *P* < 0.0001; Figure 2D). When we analyzed Keap1 protein expression, we observed a significant reduction in the ED+CP group compared with the CD+CP controls (*F*_(7,56)_ = 27.61, *P* < 0.0001; Figure 2E), whereas NP treatment significantly increased Keap1 expression in ED+NP mice compared with ED+CP mice (*P* < 0.0001). In contrast, Keap1 expression remained largely unchanged in *Nrf2^–/–^* samples (Figure 2E).

### Nrf2 peptide activates the potential antioxidant genes in alcohol ingestion in vivo.

Since Nrf2 has a regulatory role in the activation of antioxidant genes (43), in this study, we analyzed how NP treatments activate the antioxidant gene expression in alcohol-ingested mice. Immunofluorescence analysis of HO-1 expression in brain microvessels revealed a significant main effect of treatment (*F*_(2,27)_ = 58.82, *P* < 0.0001). Post hoc analysis showed a reduced expression of HO-1 in ED animals treated with CP, whereas NP activated the expression of HO-1 in ED animals in the brain microvessels (*P* < 0.0001; Figure 2, F and G). Next, we analyzed the changes in the antioxidant proteins HO-1, GPx1, and GSTm1 by Western blotting to validate the regulatory role of Nrf2 in ED-fed mice. ANOVA revealed significant main effects of treatment for all 3 proteins (HO-1: *F*_(7,88)_ = 65.56, *P* < 0.0001; GPx1: *F*_(7,88)_ = 28.18, *P* < 0.0001; GSTm1: *F*_(7,88)_ = 32.20, *P* < 0.0001) (Figure 2, H–K). Post hoc analysis showed a significant decrease in HO-1, GPx1, and GSTm1 expression in ED+CP WT animals (reduced 0.35-, 0.4-, and 0.28-fold, respectively) compared with CD+CP control WT animals (*P* < 0.0001; Figure 2, H–K). However, NP treatment in ED animals activated the expression of these 3 antioxidant proteins in comparison with CP-treated ED animals (*P* < 0.0001; Figure 2, H–K), whereas, in *Nrf2^−/−^* mice, the expression of HO-1, GPx1, and GSTm1 was not improved in NP-treated ED animals (Figure 2, H–K).

**Figure 3:**
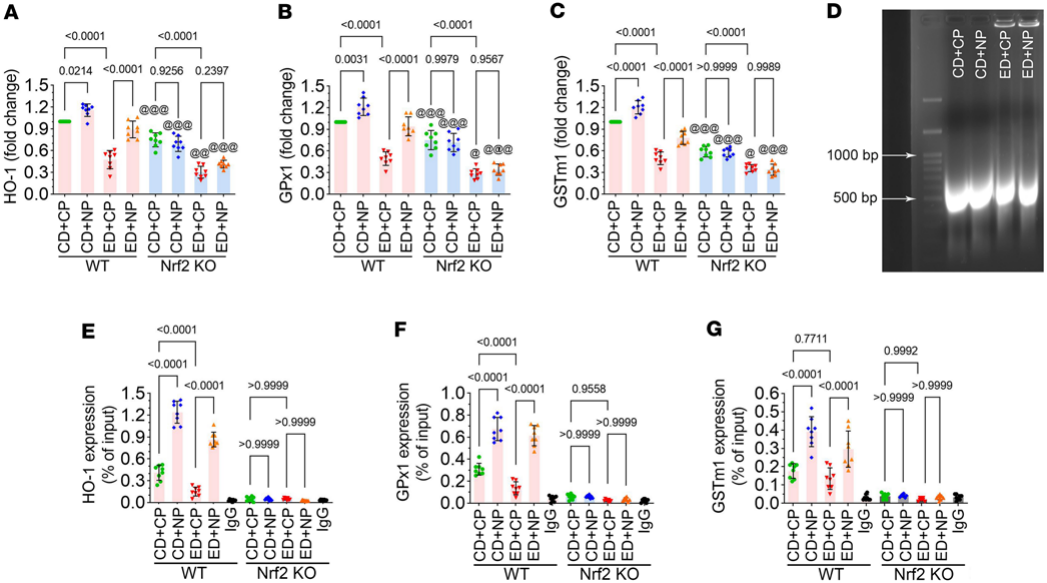
Nrf2 peptide improves the expression of antioxidant genes in alcohol ingestion. (**A**–**C**) The mRNA expression level of HO-1 (**A**), GPx1 (**B**), and GSTm1 (**C**) in the CD- and ED-fed mice of WT and *Nrf2^–/–^* groups with CP or NP treatment (*n* = 8 per group). (**D**) Agarose gel electrophoresis of purified cross-linked chromatin samples extracted from WT mouse frontal cortex. (**E**–**G**) ChIP-qPCR analysis of the DNA binding activity of HO-1 (**E**), GPx1 (**F**), and GSTm1 (**G**) in the CD- and ED-fed animals of WT and *Nrf2^–/–^* groups with CP or NP treatment (*n* = 8 per group). Anti-IgG antibody was used as a negative control. All values are expressed as mean ± SD. Statistical analysis was performed by 3-way ANOVA followed by Bonferroni’s post hoc test. *P* < 0.05 was considered statistically significant. ^@^*P* < 0.05, ^@@^*P* < 0.01, ^@@^*P* < 0.001 vs. representative groups in WT animals. Exact *P* values are shown between the compared groups.

Next, using RT-qPCR in WT and *Nrf2^−/−^* mice, we investigated the effect of alcohol on the expression of the antioxidant genes HO-1, GPx1, and GSTm1. ANOVA revealed significant main effects of treatment for all 3 genes (HO-1: *F*_(3,28)_ = 13.01, *P* < 0.0001; GPx1: *F*_(3,28)_ = 13.53, *P* < 0.0001; GSTm1: *F*_(3,28)_ = 31.65, *P* < 0.0001) (Figure 3, A–C). Post hoc analysis showed a significant reduction in the mRNA expression of HO-1, GPx1, and GSTm1 in ED+CP WT animals compared with CD+CP WT controls, and NP treatment attenuated the downregulation of these 3 antioxidant genes (*P* < 0.0001). A further decrease in the expression of HO-1, GPx1, and GSTm1 was found in ED+CP *Nrf2^−/−^* mice compared with the ED+CP WT group (at least *P <* 0.05). Moreover, NP had no effect on the expression of these 3 antioxidant genes in ED+NP *Nrf2^−/−^* mice (*P* < 0.0001; Figure 3, A–C).

Next, binding of Nrf2 to the ARE gene and activation of antioxidant genes were further confirmed by ChIP-qPCR analyses. Figure 3D shows purified cross-linked chromatin samples for ChIP-qPCR extracted from WT mouse frontal cortex. The ChIP-qPCR results indicated that both in CD+CP and in ED+CP WT mice, Nrf2 binds proximal to HO-1, GPx1, and GSTm1. ANOVA revealed significant main effects of treatment on HO-1, GPx1, and GSTm1 DNA enrichment (HO-1: *F*_(4,35)_ = 197.9, *P* < 0.0001; GPx1: *F*_(4,35)_ = 105.5, *P* < 0.0001; GSTm1: *F*_(4,35)_ = 33.07, *P* < 0.0001; Figure 3, E–G). Notably, ED+CP WT mice showed significantly reduced expression levels of HO-1, GPx1, and GSTm1 DNA compared with CD+CP WT mice (*P* < 0.0001; Figure 3, E–G). These results demonstrate that Nrf2 binds to the regulatory regions of these antioxidant genes, and alcohol reduces the expression of these antioxidant genes. As expected, *Nrf2^–/–^* mice showed a negligible expression of HO-1, GPx1, and GSTm1 in CP-treated CD and ED groups (Figure 3, E–G). Interestingly, NP-treated ED WT animals showed a significantly higher level of HO-1, GPx1, and GSTm1 genes compared with CP-treated ED WT animals, and the effect of NP was not observed in *Nrf2^–/–^* mice (*P* < 0.0001; Figure 3, E–G). This confirms that NP activates the antioxidant pathway in alcohol-ingested animals.

### Nrf2 peptide reduces the expression of oxidative stress markers.

**Figure 4:**
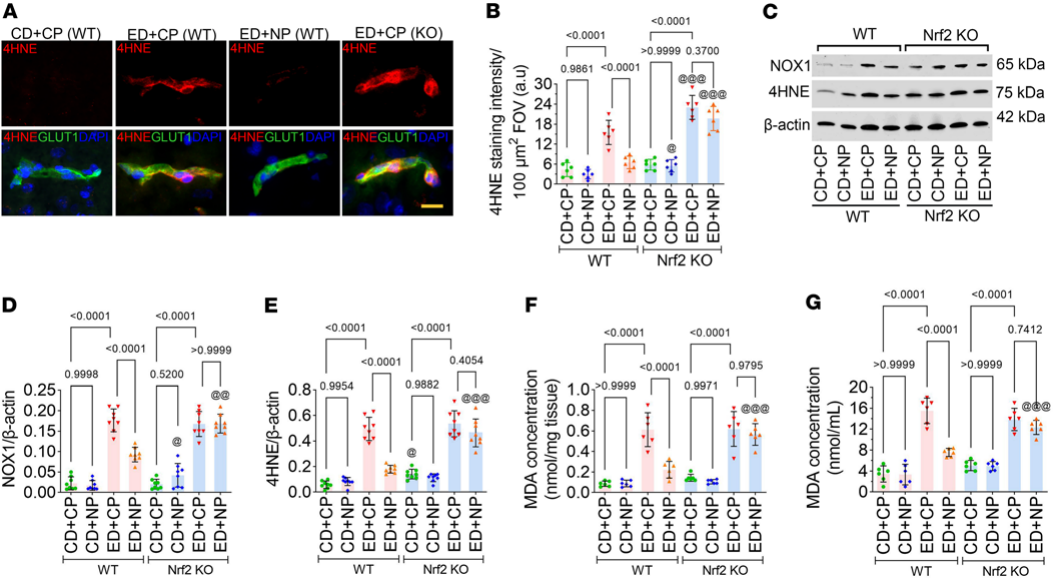
Nrf2 peptide attenuates oxidative stress. (**A** and **B**) Immunofluorescence staining of lipid peroxidation marker 4-HNE in the intact brain microvessels of mice fed with CD or ED with CP or NP treatments. Scale bar: 10 μm. (**B**) Quantification of 4-HNE staining analyzed using ImageJ software (*n* = 10 per group). (**C**–**E**) Western blot analysis of NOX1, 4-HNE, and β-actin from the mouse cortex tissue lysate of CD- and ED-fed animals in WT and Nrf2-knockout animals (*Nrf2^–/–^*) with CP or NP treatments. Bar graphs with dot plot show the ratio densitometry of NOX1 (**D**) and 4-HNE (**E**) to β-actin (*n* = 8 per group). All values are expressed as mean ± SD. (**F** and **G**) Level of MDA generation from the mouse frontal cortex tissue lysate (**F**) and blood plasma (**G**) by ELISA in CD- and ED-fed animals of WT and Nrf2 knockout groups with CP or NP treatments (*n* = 8 per group). All values are expressed as mean ± SD. Statistical analysis was performed by 3-way ANOVA, followed by Bonferroni’s post hoc test. *P* < 0.05 was considered statistically significant. ^@^*P* < 0.05, ^@@^*P* < 0.01, ^@@^*P* < 0.001 vs. representative groups in WT animals. Exact *P* values are shown between the compared groups.

Since Nrf2 regulates the expression of antioxidant genes, we aimed to investigate the effect of NP on alcohol-induced oxidative stress. In immunofluorescence staining, the expression of the lipid peroxidation marker 4-hydroxynonenal (4-HNE) was significantly higher in the brain microvessels of ED animals treated with CP (*F*_(2,15)_ = 52.65, *P* < 0.0001; Figure 4, A and B). As expected, the absence of Nrf2 in *Nrf2^–/–^* mice led to a further increase in the expression of 4-HNE in CP-treated ED groups (Figure 4, A and B). In Western blotting, statistical analysis indicated a significant effect of treatment on NADPH oxidase 1 (NOX1) and its lipid peroxidation product 4-HNE (NOX1: *F*_(3,28)_ = 14.42, *P* < 0.0001; 4-HNE: *F*_(3,28)_ = 12.51, *P* < 0.0001). Subsequent comparisons showed a significantly increased expression of NOX1 and its product 4-HNE in ED+CP WT animals compared with CD+CP WT controls (*P* < 0.0001). However, treatment with NP decreased the expression of NOX1 and 4-HNE in the ED+NP WT group, and this effect was not observed in ED+NP *Nrf2^–/–^* mice (*P* < 0.0001; Figure 4, C–E). Next, we tested the effect of alcohol and NP on the formation of the lipid peroxidation product MDA in the brain frontal cortex tissue lysate and blood plasma of CD or ED WT and *Nrf2^–/–^* mice (*F*_(3,20)_ = 5.51, *P* < 0.0001). In brain frontal cortex tissue lysates, the level of MDA was significantly increased in ED+CP WT mice compared with CD+CP WT controls (*P* < 0.0001; Figure 4F). As predicted, the level of MDA was significantly reduced in NP-treated ED WT mice. However, no significant effect of NP was observed in ED *Nrf2^–/–^* mice. A similar trend was observed in blood plasma samples (*F*_(3,20)_ = 9.36, *P* < 0.0001; Figure 4G).

### Alcohol augments the activation of the ICAM-1 signaling pathway, and Nrf2 peptide modulates it.

**Figure 5:**
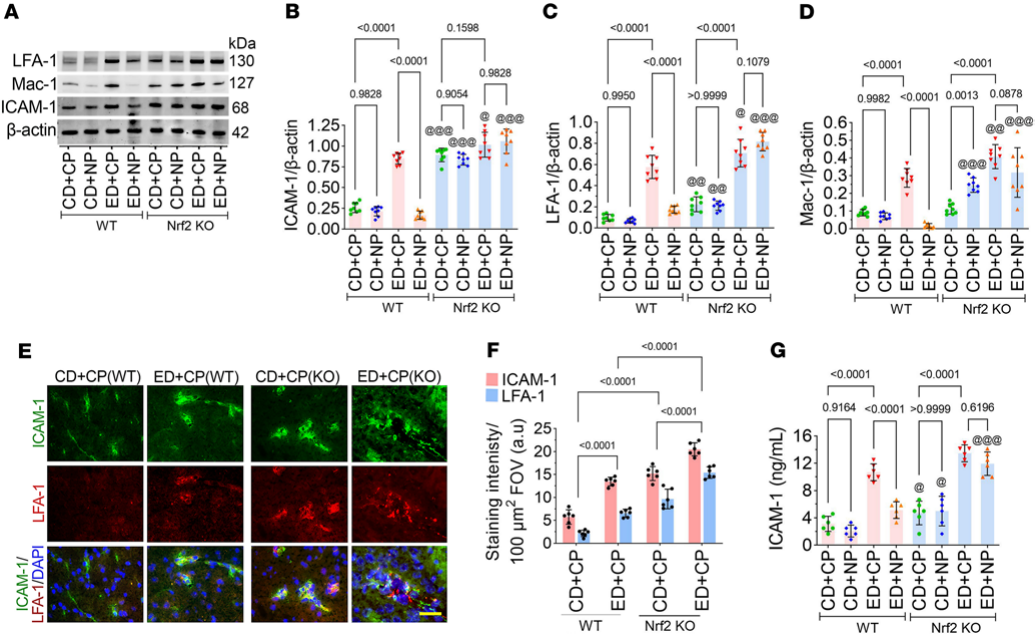
Alcohol augments the activation of ICAM-1 and its receptors LFA-1 and Mac-1. (**A**–**D**) Western blot analysis of ICAM-1, LFA-1, Mac-1, and β-actin from the mouse cortex tissue lysate of CD- and ED-fed animals in WT and *Nrf2^–/–^* with CP or NP treatments. Bar graphs with dot plot show the ratio densitometry of ICAM-1 (**B**), LFA-1 (**C**), and Mac-1 (**D**) to β-actin (*n* = 8 per group). (**E**) Immunofluorescent staining of ICAM-1 (green) merged with its receptor LFA-1 (red) and DAPI (blue) in the brain cortical tissue sections of CD- and ED-fed animals in WT and *Nrf2^–/–^* with CP or NP treatments. Scale bar: 20 μm. (**F**) Quantification of ICAM-1 and LFA-1 staining analyzed using ImageJ software (*n* = 6). (**G**) ELISA quantification of ICAM-1 in the tissue lysate of CD- and ED-fed animals in WT and *Nrf2^–/–^* with CP or NP treatments (*n* = 8 per group). All values are expressed as mean ± SD. Statistical analysis was performed by 3-way ANOVA followed by Bonferroni’s post hoc test. *P* < 0.05 was considered statistically significant. ^@^*P* < 0.05, ^@@^*P* < 0.01, ^@@^*P* < 0.001 vs. representative groups in WT animals. Exact *P* values are shown between the compared groups.

Since we observed in one of our previous studies that ICAM-1, along with its receptors LFA-1 and Mac-1, is crucial for transmigration (44), we next analyzed the effect of alcohol on this ligand and receptors and how Nrf2 regulates it. ANOVA of Western blot data showed a significant main effect of treatment on the expression of ICAM-1, LFA-1, and Mac-1 (ICAM-1: *F*_(3,28)_ = 47.88, *P* < 0.0001; LFA-1: *F*_(3,28)_ = 59.09, *P* < 0.0001; Mac-1: *F*_(3,28)_ = 36.07, *P* < 0.0001), and pairwise analysis showed significantly elevated expression of ICAM-1, LFA-1, and Mac-1 in the ED+CP group in both WT and *Nrf2^–/–^* mice compared with the CD groups of WT and *Nrf2^–/–^* mice, respectively (*P* < 0.0001; Figure 5, A–D). However, treatment with NP significantly decreased the expression of ICAM-1, LFA-1, and Mac-1 in the ED+NP WT group (*P* < 0.0001; Figure 5, A–D). NP treatment did not cause any significant change in the expression of ICAM-1, LFA-1, and Mac-1 in the ED group of *Nrf2^–/–^* mice (*P* < 0.0001; Figure 5, A–D). Immunofluorescence staining of ICAM-1 and LFA-1 showed a significant increase in their expression in ED+CP WT animals compared with the CD+CP WT group (*F*_(3,20)_ = 14.61, *P* < 0.0001; Figure 5, E and F). A further increase in the expression of ICAM-1 and LFA-1 was observed in *Nrf2^–/–^* animals (Figure 5, E and F). Next, we analyzed the level of ICAM-1 protein in blood plasma using ELISA. These results revealed a significantly increased level of ICAM-1 in the ED+CP WT group compared with the CD group of both WT and *Nrf2^–/–^* mice (*F*_(3,20)_ = 11.86, *P* < 0.0001; Figure 5G). However, NP treatment reduced ICAM-1 expression in ED+NP WT group mice. Notably, NP treatment did not alter ICAM-1 expression in the ED+NP group of *Nrf2^–/–^* mice (Figure 5G).

### Nrf2 peptide protects the brain from pericyte loss and maintains BBB integrity in alcohol ingestion.

**Figure 6:**
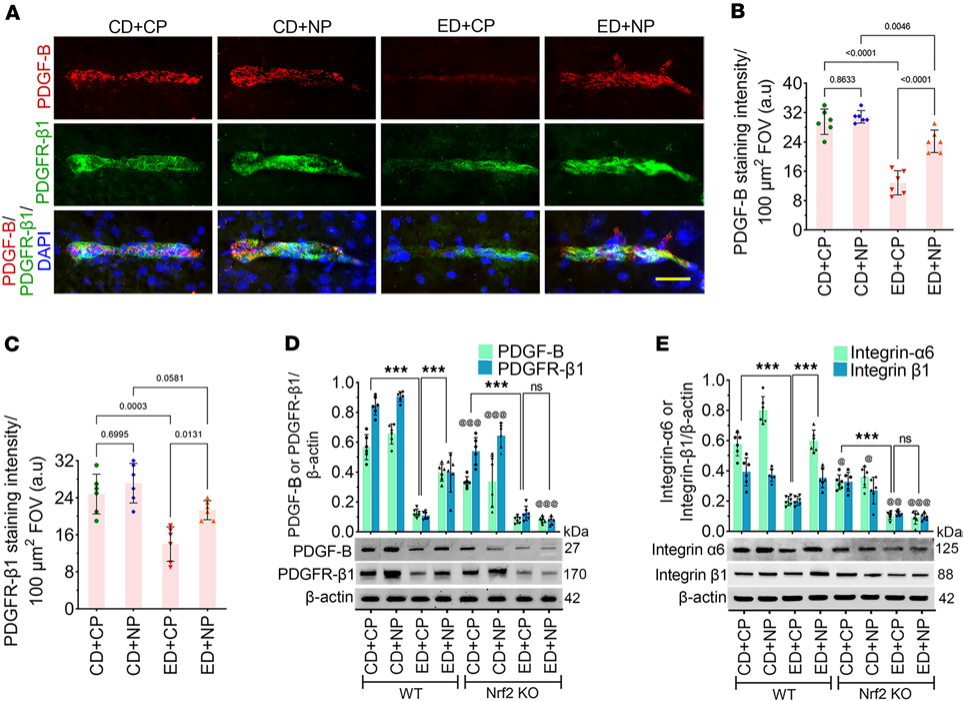
Alcohol impairs BBB integrity and Nrf2 peptide protects it. (**A**) Immunofluorescence staining of PDGF-B (red) colocalized with its receptor PDGFR-β1 (green) and merged with DAPI (blue) in the intact brain microvessels of mice fed with CD or ED with CP or NP treatments. Scale bar: 25 μm. (**B** and **C**) Quantification of PDGF-B (**B**) and PDGFR-β1 (**C**) staining analyzed using ImageJ software (*n* = 6 per group). (**D**) Western blot analysis of PDGF-B, PDGFR-β1, and β-actin from the mouse cortex tissue lysate of CD- and ED-fed animals in WT and *Nrf2^–/–^* groups with CP or NP treatments. Bar graph with dot plot shows the ratio densitometry of PDGF-B and PDGFR-β1 to β-actin (*n* = 6 per group). (**E**) Western blot analysis of integrin α_6_, integrin β_1_, and β-actin from the mouse cortex tissue lysate of CD- and ED-fed animals in WT and *Nrf2^–/–^* groups with CP or NP treatments. Bar graph with dot plot shows the ratio densitometry of integrin α_6_ and integrin β_1_ to β-actin (*n* = 6 per group). All values are expressed as mean ± SD. Statistical analysis was performed by 2-way ANOVA (**B**) and 3-way ANOVA (**C** and **D**) followed by Bonferroni’s post hoc test. *P* < 0.05 was considered statistically significant. ***P < 0.001 between the bars, as shown in the figure. ^@^*P* < 0.05, ^@@^*P* < 0.01, ^@@@^*P* < 0.001 vs. representative groups in WT animals. Exact *P* values are shown between the compared groups.

Since brain pericytes play a key role in maintaining neurovascular stability and integrity (45), we next aimed to assess the expression levels of PDGF-B (expressed in brain endothelial cells) and PDGFR-β (expressed in pericytes) to investigate how alcohol compromises pericyte function in maintaining BBB integrity, and to determine how NP protects this integrity. In double immunofluorescence, we studied the expression of PDGF-B and PDGFR-β (46, 47) in intact brain microvessels. We found that the levels of PDGF-B and PDGFR-β1 were significantly reduced in ED+CP mice compared with the CD+CP group (*P* < 0.0001; Figure 6, A–C). NP treatment protected the brain microvessels by preserving PDGF-B and PDGFR-β1 protein levels in ED+NP animals compared with ED+CP (Figure 6, A–C).

In Western blotting, using *Nrf2^−/−^* mice, we validated the role of Nrf2 in activating the PDGF-B/PDGFR-β signaling pathway. The expression levels of PDGF-B and PDGFR-β1 in ED+CP WT animals were significantly decreased (reduced to approximately one-quarter) compared with those in CD+CP WT mice (*F*_(7,48)_ = 8.48, *P* < 0.0001) (Figure 6D). As predicted, in NP-treated animals, the expression levels of PDGF-B and PDGFR-β1 were significantly increased in ED+NP WT samples compared with ED+CP WT samples (*P* < 0.0001). However, in ED+CP *Nrf2*^−/−^ mice, the expression of PDGF-B and PDGFR-β1 was further reduced in comparison with ED+CP WT animals (*P* < 0.0001), and NP treatment did not cause any change in the expression of these two proteins in ED+NP *Nrf2*^−/−^ mice (Figure 6D).

Both pericytes and endothelial cells are attached to the extracellular matrix (ECM) proteins of the basement membrane by different integrins (8, 48). To test the hypothesis that alcohol compromises the expression level of integrins and affects the integrity of the BBB and NP restores the functionality of the BBB, we analyzed the expression level of two different integrins — integrin α_6_ and integrin β_1_ — by Western blotting. The expression level of these two high–molecular weight integrins was significantly reduced in ED+CP animals compared with CD+CP control animals (reduced to one-third for integrin α_6_, and one-half for integrin β_1_; *P* < 0.0001; Figure 6E); however, no reduction was observed in the expression of these two integrins in NP-treated ED WT animals. Consistent with the genotype, in ED+NP *Nrf2*^−/−^ samples, the expression of integrin α_6_ and integrin β_1_ was not improved in comparison with ED+NP WT samples (Figure 6E).

### Nrf2 peptide ameliorates the BBB tight junction damage in alcohol ingestion.

**Figure 7:**
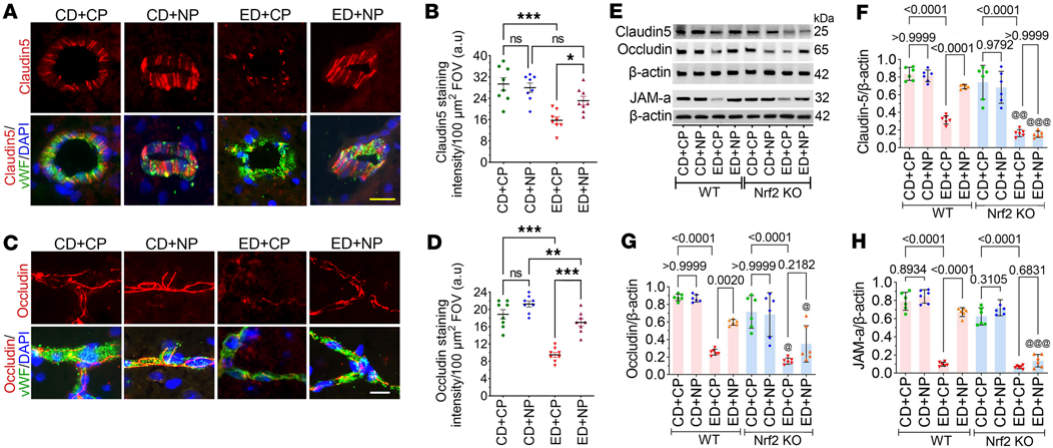
Nrf2 peptide protects the BBB in alcohol ingestion. (**A**) Immunofluorescence staining of claudin-5 (red) colocalized with vWF (green) and merged with DAPI (blue) in the cross section of intact brain microvessels of mice fed with CD or ED with CP or NP treatments. Scale bar: 25 μm. (**B**) Quantification of claudin-5 staining analyzed using ImageJ software (*n* = 8 per group). (**C**) Immunofluorescence staining of occludin colocalized with vWF (green) and merged with DAPI (blue) in the intact brain microvessels of animals fed with CD or ED with CP or NP treatments. Scale bar: 10 μm. (**E**–**H**) Western blot analysis of claudin-5, occludin, JAM-A, and β-actin from the mouse cortex tissue lysate of CD- and ED-fed animals in WT and *Nrf2^–/–^* groups with CP or NP treatments. Bar graphs with dot plot show the ratio densitometry of claudin-5 (**F**), occludin (**G**), and JAM-A (**H**) to β-actin (*n* = 8 per group). All values are expressed as mean ± SD. Statistical analysis was performed by 2-way ANOVA (**B** and **D**) and 3-way ANOVA (**F** and **G**) followed by Bonferroni’s post hoc test. Statistically significant, *P < 0.05, **P < 0.01, ***P < 0.001 vs. their representative control groups (CD) in **B** and **D**. ^@^*P* < 0.05, ^@@^*P* < 0.01, ^@@@^*P* < 0.001 vs. representative groups in WT animals. *P* < 0.05 was considered statistically significant. Exact *P* values are shown between the compared groups in **F**–**H**.

In our previous study, we demonstrated the induction of oxidative stress and BBB damage following alcohol ingestion (11), but here we demonstrate that alcohol-induced BBB damage can be repaired by treatment with NP. We studied the neuroprotective effect of NP by analyzing the BBB components as assessed by the expression level of tight junction proteins such as claudin-5 and occludin and a junctional adhesion molecule, JAM-A, in the brain microvessels of WT animals by immunostaining and Western blotting. In double immunostaining, we colocalized claudin-5 and occludin with vWF (a specific microvessel marker) to highlight the expression in brain microvessels. Variance analysis (2-way ANOVA) revealed significant main effects of alcohol treatment on claudin-5 expression in brain microvessels (*F*_(3,28)_ = 11.66, *P* < 0.0001). Follow-up pairwise comparisons showed that the expression level of claudin-5 in brain microvessel cross sections was significantly reduced (to one-half; *P* < 0.0001) in ED+CP animals compared with CD+CP mice, whereas the decrease in claudin-5 in the ED group was mitigated by NP in ED+NP mice (Figure 7, A and B). Next, when we analyzed the expression level of occludin in intact brain microvessels by immunofluorescence staining, variance analysis indicated a significant main effect of alcohol on occludin expression (*F*_(3,28)_ = 37.91; *P* < 0.0001), and the protein expression of occludin was significantly reduced in ED+CP animals compared with CD+CP controls (to one-half; *P* < 0.0001) (Figure 7, C and D). As expected, the expression of occludin protein was significantly increased (nearly restored to the level of CD+CP) in ED animals treated with NP (*P* < 0.0001; Figure 7D). Consistent with the highly branched architecture of cerebral microvessels, alcohol-induced injury resulted in discontinuous and fragmented tight junction protein staining (claudin-5 and occludin) (Figure 7, A and C), reflecting pathological disruption of microvascular integrity.

We validated the expression of these BBB proteins and the regulatory role of Nrf2 in alcohol ingestion by Western blotting. When we examined the expression level of claudin-5 and occludin in ED+CP animals, we observed a significant decrease in the expression of claudin-5 and occludin in WT mice compared with CD+CP WT animals (claudin-5: reduced from 0.88 to 0.255; *F*_(7,40)_ = 25.34, *P* < 0.0001; occludin: reduced from 0.8383 to 0.315; *F*_(7,40)_ = 47.08, *P* < 0.0001) (Figure 7, E–G). Activation of Nrf2 by NP treatment protected the expression level of claudin-5 and occludin in ED+NP WT samples compared with CP-treated ED samples (*P* < 0.0001). However, in ED+CP *Nrf2*^−/−^ mice, the expression of claudin-5 and occludin was further reduced in comparison with ED+CP WT animals (*P* < 0.0001), and NP treatment did not improve the expression of these two tight junction proteins (Figure 7, E–G). A similar trend was observed in JAM-A in Western blotting, where it was reduced to one-sixth in ED+CP WT samples compared with CD+CP WT controls (*F*_(7,40)_ = 132.2, *P* < 0.0001); however, in ED+NP *Nrf2*^−/−^ samples, the expression of JAM-A was not protected in comparison with ED+NP WT samples (Figure 7, E and H).

### Nrf2 peptide protects the brain from BBB permeability in alcohol ingestion.

**Figure 8:**
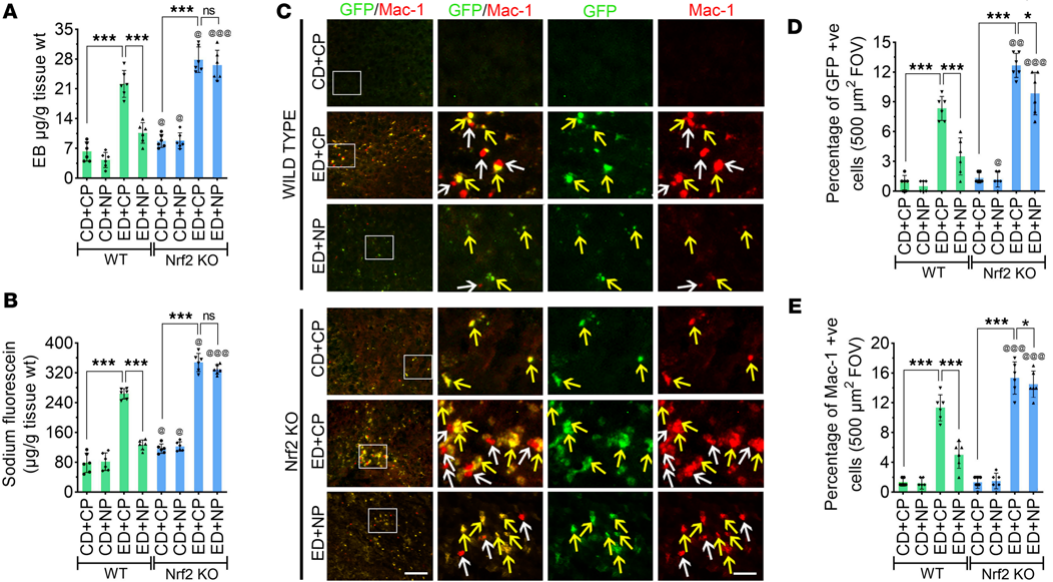
Nrf2 peptide attenuates BBB permeability and the transmigration of leukocytes to the brain in alcohol ingestion. (**A** and **B**) Graphical representation of BBB permeability showing the leakage of Evans blue (EB; 5 μM) (**A**) and sodium fluorescein (NaFl; 5 μM) (**B**) in mice fed with CD or ED with CP or NP treatments (*n* = 6 per group). (**C**) Transmigration of leukocytes was analyzed by infusion of cultured GFP monocytes (green) through the jugular vein of mice; immunostaining images show colocalization with Mac-1 (red). The second panel in each row shows selected, enlarged, and colocalized staining of GFP and Mac-1. The third and fourth panels of each row show GFP and Mac-1 staining, respectively. Scale bars: 400 μm in first panel of each row; 80 μm in second, third, and fourth panels. In second panels, the colocalized yellow cells are infused GFP cells stained with Mac-1 (yellow arrows); and red-stained cells (Mac-1 alone) are endogenous blood cells (white arrows). (**D** and **E**) Quantitative analysis of the number of GFP^+^ (**D**) and Mac-1^+^ (**E**) cells. Data are shown as mean ± SD. Statistically significant, *P < 0.05 and ***P < 0.001 vs. their representative control groups (CD) in WT and *Nrf2^–/–^*. ^@^*P* < 0.05, ^@@^*P* < 0.001, and ^@@@^*P* < 0.001 vs. representative groups in WT animals. Statistical analysis was performed by 3-way ANOVA with Bonferroni’s post hoc test.

Alcohol-induced disruption of BBB integrity was analyzed using the permeability of sodium fluorescein (NaFl) and Evans blue (EB) tracers across the BBB (7–9). Variance analysis revealed significant main effects of alcohol treatment on BBB permeability for both NaFl (MW = 376 Da; NaFl: *F*_(7,40)_ = 225.4, *P* < 0.0001) and EB (MW = 961 Da; EB: *F*_(7,40)_ = 84.65, *P* < 0.0001). Follow-up pairwise comparisons indicated that ED+CP mice showed a marked increase in the permeability of small–molecular weight NaFl (3.36-fold) and large–molecular weight tracer EB (3.5-fold) across the BBB compared with CD+CP controls (*P* < 0.0001; Figure 8, A and B). However, NP-treated ED mice exhibited a significantly reduced BBB permeability to both tracers compared with CP-treated ED animals (*P* < 0.0001), whereas all *Nrf2^–/–^* animal groups showed a significantly increased permeability of NaFl and EB compared with their respective WT animals (*P <* 0.05) (Figure 8, A and B). Moreover, as expected, NP treatment in the ED group of *Nrf2^–/–^* animals did not reduce the permeability to NaFl and EB compared with NP treatment in the ED WT group (Figure 8, A and B).

### Nrf2 peptide protects the brain from the transmigration of immune cells to the brain in alcohol ingestion.

Next, we focused on investigating the mechanisms underlying the transmigration of leukocytes into the brain after alcohol ingestion. We validated the role of Nrf2 in leukocyte transmigration using *Nrf2^–/–^* animals, and the therapeutic effect of NP was confirmed. For this experiment, we used GFP^+^ bone marrow–derived macrophages that were differentiated from monocytes isolated and cultured from GFP-transgenic mice. We infused these GFP^+^ macrophages through the jugular vein in WT or *Nrf2^–/–^* ED or CD mice with CP or NP treatments. The GFP^+^ macrophages were detected under a fluorescent microscope. We validated the transmigration of blood cells by detecting GFP^+^ cells through immunofluorescence using an anti–Mac-1 antibody (a monocyte/macrophage marker).

A 2-way ANOVA revealed significant main effects of treatment and genotype on the number of infiltrated GFP^+^ and Mac-1^+^ macrophages (GFP^+^ cells: *F*_(7,40)_ = 86.84, *P* < 0.0001; Mac-1^+^ cells: *F*_(7,40)_ = 113.1, *P* < 0.0001). Post hoc analyses showed a markedly higher number of the GFP^+^ and Mac-1^+^ macrophages in the brain tissue sections of ED+CP WT mice compared with CD+CP control mice (*P* < 0.0001; Figure 8, C–E). Interestingly, NP treatment reduced the alcohol-induced transmigration of leukocytes into the brain, and very few GFP^+^Mac-1^+^ cells were observed in the ED+NP group compared with ED+CP animals (*P* < 0.0001; Figure 8, C–E). Most of the GFP^+^ cells were colocalized with Mac-1 (yellow arrows); however, the Mac-1^+^ cells that were not colocalized with GFP (white arrows) indicated the transmigration of endogenous blood cells into the brain (Figure 8, C–E). In CP- or NP-treated ED *Nrf2^–/–^* animals, the number of GFP^+^ and Mac-1^+^ cells was significantly higher in comparison with their respective WT groups, and NP treatment had no effect in these *Nrf2^–/–^* animals (*P* < 0.0001; Figure 8, C–E). These data demonstrate that Nrf2 plays a significant role in regulating immune cell transmigration during alcohol exposure.

## Discussion

In this study, we developed a therapeutic strategy and evaluated its efficacy in protecting the brain from alcohol-induced neurovascular dysfunction and leukocyte transmigration into the brain in a mouse model of alcohol ingestion. Oxidative signaling and associated neurovascular dysfunction are the central mechanisms underlying alcohol-induced brain damage (11, 12, 14, 49). The transcription factor Nrf2 plays a crucial role in regulating oxidant resistance and cellular defense against oxidative stress. It has been implicated in protection against several chronic diseases, including neurodegenerative diseases such as Alzheimer’s and Parkinson’s diseases, traumatic brain injury, cardiovascular diseases, cancer, and metabolic disorders (18, 22, 43). Impairment of Nrf2 signaling leads to neuroinflammation, neurodegeneration, and cognitive as well as sensorimotor deficits (43, 50–52). However, the specific regulatory role of Nrf2 signaling in alcohol-induced oxidative stress–mediated BBB damage and leukocyte transmigration has not yet been elucidated. This study tested the hypothesis that alcohol-induced cerebral vascular injury and associated neurovascular complications can be repaired by activation of the antioxidant Nrf2 pathway using a small Nrf2 activator III peptide (briefly, Nrf2 peptide [NP]). NP is a synthetic peptide designed to enhance Nrf2 activation. It functions as a Keap1 inhibitor, disrupting the Keap1-Nrf2 interaction, thereby stabilizing and increasing Nrf2 levels. NP competes with Nrf2 for Keap1 binding, preventing Keap1-mediated degradation of endogenous Nrf2, which leads to cytoplasmic stabilization and accumulation of Nrf2. Once released from Keap1, Nrf2 translocates to the nucleus, where it binds to antioxidant response elements (AREs) in target gene promoters, thereby enhancing the expression of antioxidant and detoxification genes (17–20).

In recent years, peptide-based drugs have emerged as a major class of therapeutics (53), with several natural and synthetic peptides advancing toward clinical trials (54). Because alcohol use disorder (AUD) is strongly associated with oxidative stress (11, 12, 14), this study focused on an innovative peptide-based therapeutic approach targeting Nrf2 activation. Activation of endogenous Nrf2 by NP offers a clinically relevant innovative strategy for mitigating alcohol-induced oxidative damage, thereby alleviating neurovascular impairments, BBB disruption, and leukocyte transmigration into the brain. In animal studies, NP was administered daily, given the short half-life of a peptide (~20–30 minutes) (55). However, conjugation of a TAT sequence can subsequently increase the peptide’s half-life.

Oxidative stress — resulting from an imbalance between oxidants and antioxidants — is a key mechanism contributing to AUD-induced neuroinflammation and BBB dysfunction, potentially leading to physical, cognitive, and emotional deficits (11, 12, 14, 56, 57). Previously, we reported that continuous feeding of the proposed dose of ED for at least 2 weeks compromises BBB integrity and permeability in rodents (9, 56, 58). In this study, analysis of Nrf2 and p-Nrf2 expression provided insight into the activation status of the Nrf2 pathway in response to NP treatment. It is noteworthy that only faint expression of Nrf2/p-Nrf2 was detected in *Nrf2^–/–^* mice, which likely represents nonspecific antibody binding or residual, nonfunctional protein fragments, rather than active Nrf2. We also explored whether sex or alcohol consumption level modified these effects. No consistent trends were observed, and we recently examined and published these sex- and consumption-related differences (29). It is important to note that alcohol withdrawal itself represents a potent oxidative challenge, which may further modulate Nrf2 signaling. Therefore, some of the observed changes in oxidant/antioxidant balance may reflect a combined effect of chronic alcohol exposure and withdrawal-induced oxidative stress. Our results suggest that NP treatment activates Nrf2 and p-Nrf2 expression, leading to increased levels of these proteins. The nuclear localization of p-Nrf2 during stress supports the conclusion that Nrf2 is activated and translocated to the nucleus in response to alcohol-induced oxidative stress, underscoring Nrf2’s critical role in cellular stress response and antioxidant defense.

Further, in this study, the activation of antioxidant genes by NP treatment is evident in ED samples, since in CP-treated ED WT and *Nrf2^–/–^* mouse samples, the expression of antioxidant genes was highly reduced. We treated animals with NP and analyzed the activation and expression of 3 antioxidant genes, HO-1, GPx1, and GSTm1. Further, in ChIP-qPCR, we confirmed the role of Nrf2 in activating antioxidant genes. The ChIP-qPCR data are a direct indication of the efficacy of NP in activating the Nrf2 transcription factor and its linked antioxidant genes. The increased DNA pull-down in NP-treated samples reflects enhanced Nrf2 binding to antioxidant gene promoters, which likely contributes to their upregulated expression. Even though Nrf2 activates HO-1, GPx1, and GSTm1, the expression of GSTm1 is often lower, and this could be due to variations in the extent of induction depending on the gene, cell type, and oxidative stress conditions. The oxidative stress–inducing enzyme NOX1 initiates vascular injury by oxidative damage in alcohol ingestion, and this induction of NOX1 is paralleled by the signature of oxidative damage and lipid peroxidation products 4-HNE and malondialdehyde (MDA) (59, 60). We noticed that these two oxidative products were substantially high until we started NP treatments. It is obvious that NP demonstrated its efficacy in reducing the oxidative stress products after 14 days of treatment.

The key focus of this study is the investigation of the relationship between Nrf2 and ICAM-1 signaling. ICAM-1, a cell surface glycoprotein, is expressed in various cell types, including endothelial cells and immune cells like leukocytes (white blood cells) (61, 62). ICAM-1 interacts with specific integrins on the surface of leukocytes, promoting their adhesion and subsequent migration through the blood vessel wall. Two important integrins that bind to ICAM-1 are LFA-1 and Mac-1. LFA-1 is primarily found on T cells and other leukocytes, while Mac-1 is present on neutrophils, monocytes, and macrophages (63, 64). In this study, we found that NP treatment reduced the expression of ICAM-1 and its receptors LFA-1 and Mac-1 in ED samples. Additionally, data from *Nrf2^–/–^* animals confirm the role of Nrf2 in regulating the activation of ICAM-1 and its receptors LFA-1 and Mac-1 (65). Since ICAM-1 plays a key role in the transmigration of inflammatory immune cells following BBB damage, upon activating Nrf2 (through NP treatment), we observed a reduction in the transmigration of immune cells to the brain in alcohol ingestion. Our results provide strong evidence that activation of Nrf2 not only enhances the antioxidant signaling pathway but also attenuates the transmigration of inflammatory cells and prevents the inflammatory response triggered by the ICAM-1 signaling pathway.

Pericytes are important for maintaining the integrity of the BBB by interacting with endothelial cells and astrocytes, and pericyte loss is a key hallmark of BBB dysfunction (47, 66, 67). Next, we studied the impairment of PDGF-B/PDGFR-β signaling, which is crucial for the recruitment and maintenance of pericytes around endothelial cells (68, 69). Although PDGFR-β is predominantly expressed in pericytes, it is also present in other CNS cell types, including vascular smooth muscle cells, fibroblasts, and neurons (70). However, since our study primarily focuses on the brain vasculature, this limitation is unlikely to affect the interpretation of our findings. Upon binding of endothelial PDGF-B to its receptor, PDGFR-β1, found in pericytes, intracellular signaling pathways are activated. This can lead to the activation of various downstream molecules, including kinases, which in turn initiate a cascade of events within the pericytes (46). Interestingly, our results suggest that alcohol-induced reduction of PDGFR-β1 expression in pericytes has downstream effects on PDGF-B expression in endothelial cells, leading to impaired PDGF-B/PDGFR-β1 signaling. The neuroprotective role of NP in maintaining the PDGF-B/PDGFR-β1 signaling was evident in our data, and activation of Nrf2 by NP helps maintain BBB integrity during alcohol ingestion. Next, we demonstrated how Nrf2 facilitates the interaction between endothelial cells and pericytes by examining the expression of two integrins, integrin α_6_ and integrin β_1_, which play a crucial role in linking these two cell types (71). Here, we present evidence of integrin-mediated interactions between endothelial cells, or pericytes, and the ECM, highlighting the role of the NP in maintaining BBB integrity and regulating its permeability. While changes in integrin expression are associated with BBB dysfunction (8), we acknowledge that these integrins are expressed in multiple cell types, and our analyses were performed on whole-tissue lysates. Therefore, the observed alterations in integrin expression likely reflect changes across the neurovascular unit rather than being specific to endothelial cell–pericyte interactions. Consistently, NP protects the brain from BBB compromise resulting from pericyte loss and integrin impairment during alcohol ingestion. Although some biochemical analyses were performed using whole cortical lysates and may include non-endothelial contributions, microvascular specificity is strongly supported by the use of established microvessel-associated markers, including PDGFR-β, PDGF-B, integrins, and tight junction proteins, which are enriched in vascular and perivascular compartments. Future studies employing isolated microvessels or endothelial cell–specific Nrf2 conditional knockout models will further refine cell-type resolution.

We further established the connection between loss of pericyte-endothelium integrity and increased BBB permeability by validating the detection of NaFl/EB tracers, which provided evidence of enhanced BBB permeability in alcohol ingestion. Activation of Nrf2 by NP treatment attenuated the BBB permeability and leakage. The adhesion of leukocytes to endothelial cells is a critical stage in the migration of leukocytes into injured tissues (72). This adhesion is regulated in part by interactions between ICAM-1 on endothelial cells and a group of glycoproteins such as CD11a/CD18 (LFA-1) and/or CD11b/CD18 (Mac-1) on leukocytes (73). Therefore, activation of Nrf2 via NP treatment suppressed the ICAM-1 signaling via LFA-1 and Mac-1, and this represents a valuable approach for attenuating the transmigration of immune cells in alcohol ingestion. In the validation of transmigration of leukocytes to the brain, we infused GFP leukocytes and analyzed their presence in the brain. While GFP signal can degrade over time and during tissue processing, we validated transmigration of exogenous leukocytes by costaining with the Mac-1 antibody to distinguish them from endogenous cells, and they appear in yellow in Figure 8C. We acknowledge that some endogenous Mac-1^+^ cells could include exogenous cells with reduced GFP signal. Here, we investigated the mechanisms of alcohol-mediated transmigration of leukocytes and explored the potential therapeutic scope of NP in mitigating alcohol-mediated transmigration of leukocytes via LFA-1 and Mac-1. The results obtained in *Nrf2^–/–^* animals validated the role of Nrf2 in the downregulation of the ICAM-1 pathway, and in WT animals, NP treatment reduced the transmigration of leukocytes to the brain in alcohol ingestion.

## Methods

### Sex as a biological variable.

Our study used both male and female adult mice. Therefore, the findings are expected to be relevant to both males and females, although no experiments were performed to test for differences between the sexes.

### Animals, alcohol diet, and peptide treatments.

For this study, 10-week-old male and female C57BL/6 wild-type (WT) and Nrf2-knockout (*Nrf2^–/–^*, C57BL/6 strain) mice were used (The Jackson Laboratory). The animals were housed in sterile cages in a temperature-controlled, pathogen-free animal facility on a 12-hour light/12-hour dark cycle. The study was conducted on 8 experimental groups of animals, comprising 4 treatment regimens each in WT and *Nrf2^–/–^* mice. The treatments were (a) CD+CP (control diet + control peptide), (b) CD+NP (control diet + Nrf2 peptide), (c) ED+CP (EtOH diet + control peptide), and (d) ED+NP (EtOH diet + Nrf2 peptide). Four weeks of alcohol treatment was administered by feeding of Lieber-DeCarli liquid diet supplemented with absolute EtOH (~5% vol/vol) in 1 L (5.35 kcal/g) (Dyets Inc.) (9, 56, 58, 74, 75). Concurrently, control animals were pair-fed with the Lieber-DeCarli CD. Pair-fed control animals were included to match the caloric intake of the ethanol-fed group, ensuring that observed effects were due to ethanol exposure rather than differences in food consumption or nutrition. Neither additional liquid nor water was provided to the animals, because they were supplied with a liquid diet, and the amount of liquid diet that each animal consumed each day was measured (9, 11, 58). The dosing was not adjusted to individual body weight, as all animals were of the same age and showed similar body weights (±1.6 g) at the start of the experiment. Blood alcohol concentrations and withdrawal-related effects following 28 days of ethanol exposure have been characterized in our previous study (29), confirming that this regimen produces physiologically relevant ethanol levels and mild dependence without severe withdrawal.

From day 29 onward, the NP was administered subcutaneously at 100 μg/100 μL in 0.9% saline (1 injection daily). We chose this peptide dose by conducting a dose-response study to ensure safety and nontoxicity. An equal volume of scrambled TAT peptide (briefly, control peptide [CP]) was injected in CD and ED mice. The detailed experimental procedure is given in the online supplemental material.

### RT-qPCR and ChIP-qPCR.

The mRNA expression levels of HO-1, GPx1, GSTm1, NQO1, Nrf2, and GAPDH were determined by RT-qPCR using the primers listed in Supplemental Table 1. Using the RNeasy Mini kit (QIAGEN, catalog 74104), total RNA was extracted from the animal brain frontal cortex tissue per the manufacturer’s instructions. The detailed experimental procedures for RT-qPCR and ChIP-qPCR are given in Supplemental Methods.

### Immunofluorescence and microscopy.

Coronal brain sections (10 μm) were fixed with 4% paraformaldehyde, blocked in 3% goat serum plus 0.1% Triton X-100, and incubated overnight at 4°C with primary antibodies against HO-1, GPx1, GSTm1, claudin-5, occludin, Mac-1, PDGF-B, or PDGFR-β1. For colocalization, anti-GLUT1 or -vWF was used. Alexa Fluor 488/594 secondary antibodies (1:500) were applied for 1 hour, and nuclei were counterstained with DAPI. Images were captured using a Leica DMi8 microscope and quantified with ImageJ (NIH) under identical acquisition settings. The detailed experimental procedures are given in Supplemental Methods.

### Western blotting.

Frontal cortex tissue (~50 mg) was lysed in Cell Lytic-M buffer (Sigma-Aldrich, catalog C2978) with protease inhibitors. Equal protein (15 μg) was separated by SDS-PAGE, transferred to nitrocellulose, and probed with specific primary and HRP-conjugated secondary antibodies. Bands were visualized by chemiluminescence (Advansta) and analyzed using ImageJ, normalized to β-actin. The detailed experimental procedures are given in Supplemental Methods.

### ELISA.

Using specific ELISA kits, the levels of Nrf2 (catalog LS-F2192-1, LS Bio) and ICAM-1 (ab203884, Abcam) were analyzed in blood plasma and tissue lysate per the manufacturer’s instructions.

### In vivo BBB permeability assay.

The effect of ethanol on BBB permeability was studied in animal models using the sodium fluorescein (NaFl) and Evans blue (EB) tracer dye mixtures (5 μM each) as previously reported (7, 8, 76). The detailed experimental procedure is given in Supplemental Methods.

### Transmigration assay in vivo.

We followed our well-established method for in vivo transmigration assay (9, 62). The detailed experimental procedure is given in Supplemental Methods.

### Use of generative artificial intelligence.

The graphical abstract was created with ChatGPT (OpenAI; GPT-5.2) on January 29, 2026, using author-provided study details; prompts included “generate a schematic figure illustrating oxidative stress, BBB disruption, and peptide-mediated inhibition” and “describe Nrf2-activated peptide treatment inducing Nrf2 activation and suppression of oxidative stress and BBB damage in alcohol-induced injury.” The authors reviewed and edited the output.

### Statistics.

Sample sizes were prospectively derived by power analyses using G*Power (University of Dusseldorf, Dusseldorf, Germany) based on our previous observations with outcome variations and effect sizes in the mouse model (8, 43, 44, 77, 78). Our sample sizes were determined with the condition of an 80% chance of detecting a moderate effect size. GraphPad Prism v9 was used for the statistical analysis of data. The data were tested for normality and equality of variance and analyzed using unpaired 2-tailed *t* tests given that our data were collected from independent groups. Interactions between samples/groups were analyzed by 3-way ANOVA (since there were 3 independent variables: liquid diet, genotype, and peptide treatment) followed by Bonferroni’s post hoc test. Data are expressed as mean ± SD, and *P* < 0.05 was considered as statistical significance. GAPDH was used as the housekeeping gene for normalization in RT-PCR, and gene expression levels were calculated relative to the CD+CP control group. Western blot data were quantified by densitometry analysis using ImageJ software (29, 62, 79) normalized to β-actin. The immunostaining intensity or number of positive cells was quantified in ImageJ by application of uniform threshold levels and measurement of mean fluorescence intensity or positive cell count within defined regions of interest, without conversion of images to binary format (29, 62, 79). We used a double-blinded study design whereby mice were assigned a unique subject number and then randomized to treatment groups in a predetermined manner by a blinded study coordinator. Blinded investigators performed all data acquisition of outcome measures. After the final data acquisition, mice were decoded, and final analyses were performed.

### Study approval.

All the experiments were performed in accordance with institutional ethical guidelines for laboratory animal care established by the National Institutes of Health and the Seton Hall University Institutional Animal Care and Use Committee at the JFK University Medical Center (Edison, New Jersey, USA).

### Data and materials availability.

The complete dataset generated in this study is described and provided in this article and in the supplemental material. Values for all data points in graphs are reported in the Supporting Data Values file. The data and materials that support the findings of this study are also available upon reasonable request. A more detailed description of the materials and methods used is provided in Supplemental Methods.

## Author contributions

BBS, YAP, ZK, and SA carried out the experiments and performed the data acquisition. PMAM designed the project, supervised the execution of the experiments, interpreted and analyzed the data, and wrote the manuscript. All authors read and approved the final manuscript.

## Funding support

This work is the result of NIH funding, in whole or in part, and is subject to the NIH Public Access Policy. Through acceptance of this federal funding, the NIH has been given a right to make the work publicly available in PubMed Central.

- NIH grants 5R21AA030625 and 5R01NS133233 to PMAM.

## Supplementary Material

Supplemental data

Unedited blot and gel images

Supporting data values

## Version 1 02/17/2026

In-Press Preview

## Version 2 03/23/2026

Electronic publication

## References

[1] Persidsky Y (2006). Blood-brain barrier: structural components and function under physiologic and pathologic conditions. J Neuroimmune Pharmacol.

[2] Kanmogne GD (2005). HIV-1 gp120 proteins alter tight junction protein expression and brain endothelial cell permeability: implications for the pathogenesis of HIV-associated dementia. J Neuropathol Exp Neurol.

[3] Persidsky Y (2006). Rho-mediated regulation of tight junctions during monocyte migration across the blood-brain barrier in HIV-1 encephalitis (HIVE). Blood.

[4] Padden M (2007). Differences in expression of junctional adhesion molecule-A and beta-catenin in multiple sclerosis brain tissue: increasing evidence for the role of tight junction pathology. Acta Neuropathol.

[5] Van Sorge NM, Doran KS (2012). Defense at the border: the blood-brain barrier versus bacterial foreigners. Future Microbiol.

[6] Cheng T (2006). Activated protein C inhibits tissue plasminogen activator-induced brain hemorrhage. Nat Med.

[7] Muneer PMA (2013). Induction of oxidative and nitrosative damage leads to cerebrovascular inflammation in an animal model of mild traumatic brain injury induced by primary blast. Free Radic Biol Med.

[8] Bhowmick S (2019). Impairment of pericyte-endothelium crosstalk leads to blood-brain barrier dysfunction following traumatic brain injury. Exp Neurol.

[9] Muneer PMA (2012). The mechanisms of cerebral vascular dysfunction and neuroinflammation by MMP-mediated degradation of VEGFR-2 in alcohol ingestion. Arterioscler Thromb Vasc Biol.

[10] Haorah J (2007). Oxidative stress activates protein tyrosine kinase and matrix metalloproteinases leading to blood-brain barrier dysfunction. J Neurochem.

[11] Alikunju S (2011). The inflammatory footprints of alcohol-induced oxidative damage in neurovascular components. Brain Behav Immun.

[12] Haorah J (2005). Alcohol-induced oxidative stress in brain endothelial cells causes blood-brain barrier dysfunction. J Leukoc Biol.

[13] Rump TJ (2010). Acetyl-L-carnitine protects neuronal function from alcohol-induced oxidative damage in the brain. Free Radic Biol Med.

[14] Haorah J (2008). Mechanism of alcohol-induced oxidative stress and neuronal injury. Free Radic Biol Med.

[15] Moi P (1994). Isolation of NF-E2-related factor 2 (Nrf2), a NF-E2-like basic leucine zipper transcriptional activator that binds to the tandem NF-E2/AP1 repeat of the beta-globin locus control region. Proc Natl Acad Sci U S A.

[16] Muneer PMA (2023). Nrf2 as a potential therapeutic target for traumatic brain injury. J Integr Neurosci.

[17] Kobayashi M, Yamamoto M (2005). Molecular mechanisms activating the Nrf2-Keap1 pathway of antioxidant gene regulation. Antioxid Redox Signal.

[18] Itoh K (1997). An Nrf2/small Maf heterodimer mediates the induction of phase II detoxifying enzyme genes through antioxidant response elements. Biochem Biophys Res Commun.

[19] Hayashi A (2003). Transcription factor Nrf2 is required for the constitutive and inducible expression of multidrug resistance-associated protein 1 in mouse embryo fibroblasts. Biochem Biophys Res Commun.

[20] Chen S (2005). Transforming growth factor-beta1 increases CXCR4 expression, stromal-derived factor-1alpha-stimulated signalling and human immunodeficiency virus-1 entry in human monocyte-derived macrophages. Immunology.

[21] Bhakkiyalakshmi E (2015). The emerging role of redox-sensitive Nrf2-Keap1 pathway in diabetes. Pharmacol Res.

[22] Ma Q (2013). Role of Nrf2 in oxidative stress and toxicity. Annu Rev Pharmacol Toxicol.

[23] Xiong W (2015). NRF2 promotes neuronal survival in neurodegeneration and acute nerve damage. J Clin Invest.

[24] Kaidery NA (2013). Targeting Nrf2-mediated gene transcription by extremely potent synthetic triterpenoids attenuate dopaminergic neurotoxicity in the MPTP mouse model of Parkinson’s disease. Antioxid Redox Signal.

[25] Alibhai FJ (2014). Short-term disruption of diurnal rhythms after murine myocardial infarction adversely affects long-term myocardial structure and function. Circ Res.

[26] Lamle J (2008). Nuclear factor-eythroid 2-related factor 2 prevents alcohol-induced fulminant liver injury. Gastroenterology.

[27] Wu KC (2012). Role of Nrf2 in preventing ethanol-induced oxidative stress and lipid accumulation. Toxicol Appl Pharmacol.

[28] Jensen JS (2013). Alcohol causes alveolar epithelial oxidative stress by inhibiting the nuclear factor (erythroid-derived 2)-like 2-antioxidant response element signaling pathway. Am J Respir Cell Mol Biol.

[29] Bhowmick S (2022). NADPH oxidase-induced activation of transforming growth factor-beta-1 causes neuropathy by suppressing antioxidant signaling pathways in alcohol use disorder. Neuropharmacology.

[30] Feng J (2023). Oxidative stress, the blood-brain barrier and neurodegenerative diseases: the critical beneficial role of dietary antioxidants. Acta Pharm Sin B.

[31] Suchankova P (2015). The glucagon-like peptide-1 receptor as a potential treatment target in alcohol use disorder: evidence from human genetic association studies and a mouse model of alcohol dependence. Transl Psychiatry.

[32] Ohtake Y (2014). The effect of systemic PTEN antagonist peptides on axon growth and functional recovery after spinal cord injury. Biomaterials.

[33] GrandPre T (2002). Nogo-66 receptor antagonist peptide promotes axonal regeneration. Nature.

[34] Craik DJ (2013). The future of peptide-based drugs. Chem Biol Drug Des.

[35] Fosgerau K, Hoffmann T (2015). Peptide therapeutics: current status and future directions. Drug Discov Today.

[36] Steel R (2012). Anti-inflammatory effect of a cell-penetrating peptide targeting the Nrf2/Keap1 interaction. ACS Med Chem Lett.

[37] Inoyama D (2012). Optimization of fluorescently labeled Nrf2 peptide probes and the development of a fluorescence polarization assay for the discovery of inhibitors of Keap1-Nrf2 interaction. J Biomol Screen.

[38] Schwarze SR (1999). In vivo protein transduction: delivery of a biologically active protein into the mouse. Science.

[39] Madani F (2011). Mechanisms of cellular uptake of cell-penetrating peptides. J Biophys.

[40] Toro A, Grunebaum E (2006). TAT-mediated intracellular delivery of purine nucleoside phosphorylase corrects its deficiency in mice. J Clin Invest.

[41] Stalmans S (2015). Cell-penetrating peptides selectively cross the blood-brain barrier in vivo. PLoS One.

[42] Zou LL (2013). Cell-penetrating peptide-mediated therapeutic molecule delivery into the central nervous system. Curr Neuropharmacol.

[43] Bhowmick S (2019). Traumatic brain injury-induced down regulation of Nrf2 activates inflammatory response and apoptotic cell death. J Mol Med (Berl).

[44] Bhowmick S (2021). Intercellular adhesion molecule-1-induced posttraumatic brain injury neuropathology in the prefrontal cortex and hippocampus leads to sensorimotor function deficits and psychological stress. eNeuro.

[45] Daneman R (2010). Pericytes are required for blood-brain barrier integrity during embryogenesis. Nature.

[46] Arimura K (2012). PDGF receptor β signaling in pericytes following ischemic brain injury. Curr Neurovasc Res.

[47] Armulik A (2010). Pericytes regulate the blood-brain barrier. Nature.

[48] Stratman AN (2009). Pericyte recruitment during vasculogenic tube assembly stimulates endothelial basement membrane matrix formation. Blood.

[49] Wang J (2013). Oxidation of ethanol in the rat brain and effects associated with chronic ethanol exposure. Proc Natl Acad Sci U S A.

[50] Yao W (2016). Role of Keap1-Nrf2 signaling in depression and dietary intake of glucoraphanin confers stress resilience in mice. Sci Rep.

[51] Yang T (2020). Ischemic preconditioning provides long-lasting neuroprotection against ischemic stroke: the role of Nrf2. Exp Neurol.

[52] Dinkova-Kostova AT (2018). The role of Nrf2 signaling in counteracting neurodegenerative diseases. FEBS J.

[53] Vlieghe P (2010). Synthetic therapeutic peptides: science and market. Drug Discov Today.

[54] Mandal SM (2014). Challenges and future prospects of antibiotic therapy: from peptides to phages utilization. Front Pharmacol.

[55] Mathur D (2016). PEPlife: a repository of the half-life of peptides. Sci Rep.

[56] Muneer PMA (2017). Activation of NLRP3 inflammasome by cholesterol crystals in alcohol consumption induces atherosclerotic lesions. Brain Behav Immun.

[57] Harper C (1998). The neuropathology of alcohol-specific brain damage, or does alcohol damage the brain?. J Neuropathol Exp Neurol.

[58] Muneer PMA (2011). Inhibitory effects of alcohol on glucose transport across the blood–brain barrier leads to neurodegeneration: preventive role of acetyl-l-carnitine. Psychopharmacology (Berl).

[59] Lu XY (2014). NADPH oxidase inhibition improves neurological outcome in experimental traumatic brain injury. Neurochem Int.

[60] Tavazzi B (2005). Cerebral oxidative stress and depression of energy metabolism correlate with severity of diffuse brain injury in rats. Neurosurgery.

[61] de Fougerolles AR (1994). Characterization of the function of intercellular adhesion molecule (ICAM)-3 and comparison with ICAM-1 and ICAM-2 in immune responses. J Exp Med.

[62] Saikia BB (2024). ICAM-1 deletion using CRISPR/Cas9 protects the brain from traumatic brain injury-induced inflammatory leukocyte adhesion and transmigration cascades by attenuating the Paxillin/FAK-dependent Rho GTPase pathway. J Neurosci.

[63] Henderson RB (2001). The use of lymphocyte function-associated antigen (LFA)-1-deficient mice to determine the role of LFA-1, Mac-1, and alpha4 integrin in the inflammatory response of neutrophils. J Exp Med.

[64] Romanova LY, Mushinski JF (2011). Central role of paxillin phosphorylation in regulation of LFA-1 integrins activity and lymphocyte migration. Cell Adh Migr.

[65] Frank PG, Lisanti MP (2008). ICAM-1: role in inflammation and in the regulation of vascular permeability. Am J Physiol Heart Circ Physiol.

[66] Sweeney MD (2016). Pericytes of the neurovascular unit: key functions and signaling pathways. Nat Neurosci.

[67] Bell RD (2010). Pericytes control key neurovascular functions and neuronal phenotype in the adult brain and during brain aging. Neuron.

[68] Bjarnegard M (2004). Endothelium-specific ablation of PDGFB leads to pericyte loss and glomerular, cardiac and placental abnormalities. Development.

[69] Enge M (2002). Endothelium-specific platelet-derived growth factor-B ablation mimics diabetic retinopathy. EMBO J.

[70] Armulik A (2011). Pericytes: developmental, physiological, and pathological perspectives, problems, and promises. Dev Cell.

[71] Albelda SM (1989). Identification and characterization of cell-substratum adhesion receptors on cultured human endothelial cells. J Clin Invest.

[72] Nourshargh S, Alon R (2014). Leukocyte migration into inflamed tissues. Immunity.

[73] Rautou PE (2011). Microparticles from human atherosclerotic plaques promote endothelial ICAM-1-dependent monocyte adhesion and transendothelial migration. Circ Res.

[74] Dong Y (2020). Metabolomics study of the hepatoprotective effect of Phellinus igniarius in chronic ethanol-induced liver injury mice using UPLC-Q/TOF-MS combined with ingenuity pathway analysis. Phytomedicine.

[75] Lieber CS, DeCarli LM (1986). The feeding of ethanol in liquid diets. Alcohol Clin Exp Res.

[76] Hawkins BT, Egleton RD (2006). Fluorescence imaging of blood-brain barrier disruption. J Neurosci Methods.

[77] Patel RK (2017). Transforming growth factor-beta 1 signaling regulates neuroinflammation and apoptosis in mild traumatic brain injury. Brain Behav Immun.

[78] Bhowmick S (2018). Synergistic inhibition of ERK1/2 and JNK, not p38, phosphorylation ameliorates neuronal damages after traumatic brain injury. Mol Neurobiol.

[79] Muneer PMA (2022). Synergistic effect of mild traumatic brain injury and alcohol aggravates neuroinflammation, amyloidogenesis, tau pathology, neurodegeneration, and blood-brain barrier alterations: Impact on psychological stress. Exp Neurol.

