# Supplementary material for: Nrf2 activator peptide protects the brain from cerebral vascular dysfunction in alcohol ingestion
Source: JCI Insight. 2026 Feb 17;11(6):e188004. doi: 10.1172/jci.insight.188004 (PMC13043087; doi:10.1172/jci.insight.188004)
Supplement: Supplemental data [file jciinsight-11-188004-s023.pdf]

Nrf2 activator peptide protects the brain from cerebral vascular dysfunction in alcohol ingestion.

Bibhuti Ballav Saikia[3], Saleena Alikunju[2], Yemin A. Poovanthodi[3], Zayan Kassim[3], P. M. Abdul Muneer[1,3]*

Affiliation:

1. Laboratory of CNS injury and Molecular Therapy, Department of Biomedical Engineering, Florida International University, 10555 West Flager Street, Miami, FL-33174, United States.
2. Miami Project to Cure Paralysis, Lois Pope LIFE Center, Miller School of Medicine, University of Miami, 1095 NW 14th Terrace, Miami, FL 33136, United States.
3. JFK Neuroscience Institute, Hackensack Meridian Health JFK University Medical Center, 65 James St, Edison, NJ-08820, United States.

***Corresponding author:**
P. M. A. Muneer,
Laboratory of CNS Injury and Molecular Therapy,
Department of Biomedical Engineering,
Florida International University,
10555 West Flager Street, Miami, FL-33174.
Phone: 305-348-0187
Email: mmuneer@fiu.edu

Supplemental methods

Assessment of Drug Safety and Effectiveness

To evaluate the safety and effectiveness of the Nrf2 peptide (NP), a dose-response study was first conducted in human brain microvascular endothelial cells (hBMVECs) using 0-150 µM of NP (i.e., 0, 10, 25, 50, 75, 100, 125, 150 µM). Cell viability was determined using the MTT assay, and apoptosis was assessed by Annexin V Western blot analysis. The 50 µM concentration produced maximal cell survival and minimal apoptotic activity in cells. For *in vivo* studies, NP was administered at 100 µg per mouse (4 mg/kg in 100 µL). Although the *in vitro* concentration was lower, the higher *in vivo* dose accounts for systemic distribution, dilution in tissues, and metabolic degradation to ensure effective concentrations at the target site. In treated animals, safety was monitored by recording body weight, food intake, grooming, and general activity throughout the experimental period. Drug effectiveness was evaluated by measuring blood-brain barrier (BBB) permeability (Na-Fl and Evans Blue assays), tight-junction protein expression (ZO-1, claudin-5, occludin), and oxidative stress markers (NOX1, 4HNE, MDA, and ROS analysis), which collectively reflect BBB integrity and neuroprotection.

Animals, alcohol diet, and peptide treatments

For this study, ten-week-old male and female C57BL/6 wild-type (WT) and Nrf2 knockout (Nrf2 KO, *Nrf2*$^{-/-}$, C57BL/6 strain) mice were used (Jackson Laboratory, Bar Harbor, ME). The animals

were housed in sterile cages in temperature-controlled, pathogens-free, and a 12-hour light/dark cycle animal facility. All the experiments were performed in accordance with institutional ethical guidelines for laboratory animal care established by the National Institutes of Health and the Seton Hall University Institutional Animal Care and Use Committee (IACUC) at the JFK University Medical Center in Edison, New Jersey. The study was conducted in 8 experimental groups of animals, comprising 4 treatment regimens each in WT and *Nrf2*$^{-/-}$ mice. The treatments are 1) CD+CP (control diet+control peptide), 2) CD+NP (control diet+Nrf2 peptide), 3) ED+CP (EtOH Diet+control peptide), and 4) ED+NP (EtOH diet+Nrf2 peptide). Four weeks of alcohol treatment were administered by feeding LieberDeCarli liquid diet supplemented with absolute EtOH (approx 5% v/v) in 1L (5.35 kcal/gm) (Dyets Inc, Bethlehem, PA)[1-5]. Concurrently, control animals were pair-fed with the LieberDeCarli CD. Neither additional liquid nor water was provided to the animals because they were supplied with a liquid diet, and the amount of liquid diet that each animal consumed each day was measured[4-6].

From Day 29 onwards, the NP was administered subcutaneously (SQ) at 100 µg/100 µL in 0.9% saline (daily one injection). We chose this peptide dose by conducting a dose-response study to ensure safety and non-toxicity. An equal volume of scrambledTAT peptide (briefly, control peptide (CP)) was injected in CD and ED mice. Blood samples were collected from the tail vein using a 30 G 1/2 needle on Day 0 (baseline), 14, 28, 42. After 2 weeks of NP or CP treatment, on Exp Day 42, animals were anesthetized (80 mg/kg Ketamine and 10 mg/kg Xylazine; each at 100 mg/ml) and perfused transcardially with 150 mL of 1X PBS and 100 mL of 4% paraformaldehyde (PFA). Then the brains were harvested, fixed in 4% PFA, and embedded in OCT for immunostaining (in ten µm coronal cryostat sections). The frontal cortex was isolated by carefully removing the olfactory bulbs and making a coronal section approximately 1.5–2.0 mm posterior to bregma, followed by dissecting the cortical tissue from both hemispheres while avoiding deeper structures such as the striatum and corpus callosum[7]. We used non-perfused animals' brains and microvessels for protein and mRNA extraction for RT-qPCR, western blotting, immunoprecipitation, ChiP-qPCR, and ELISA as per our well-established protocols[3,8-11]. The standard MCDB protocol was followed for the isolation of brain microvessels[12,13]. Briefly, the tissue homogenization was performed in 8 mL complete MCDB supplemented with 2% fetal bovine serum, 100 UI/mL penicillin, 100µg/mL streptomycin, and 0.25 µg/mL fungizone using a Dounce homogenizer. After centrifugation at 2,000g for 5 min at 4°C, the pellet was resuspended in 8 mL of 15% (wt/vol) dextran–DPBS. Then the suspension was centrifuged at 10,000g for 15 min at 4°C. The pellet (a red, microvessel-containing pellet) was dissolved in 1 mL DPBS, transferred to a 40-µm cell strainer, and washed through with <10 mL of DPBS. The pellet was suspended in 200 µL sterile PBS buffer, and frozen at –80°C until analysis.

Reverse transcriptase-quantitative PCR (RT-qPCR)

The mRNA expression levels of HO-1, GPx1, GSTm1, NQO1, Nrf2, and GAPDH were determined by RT-qPCR. Using the Qiagen RNeasy Mini kit (Qiagen, Cat. No. 74104), total RNA was extracted from the animal brain frontal cortex tissue as per the manufacturer's instructions. To eliminate genomic DNA contamination, DNase I treatment was applied for 20 minutes at 37ºC. RNA quantity, purity, and integrity were assessed using Qubit 2.0 (Thermo Fisher) and Agilent Bioanalyzer (Agilent Technologies). cDNA was synthesized from the total RNA using an iScript cDNA synthesis kit (Biorad, USA). Primer sequences are given in Table 1 below. Using 25 ng of cDNA, gene-specific forward and reverse primers, and an iTaq Universal (Bio-Rad Laboratories, USA) in a 20 µl reaction on a StepOne Real-Time System (Applied Biosystems, USA) under standard cycle conditions, PCR amplification was carried out. GAPDH mRNA was used as an

endogenous control. The fold-change differences between groups were calculated using the ΔΔCt method.

Chromatin immunoprecipitation-qPCR Assay

Brain tissue was extracted from WT and *Nrf2*$^{-/-}$ mice and crosslinked with 1% formaldehyde at room temperature, and subsequently quenched with glycine. After washing with 1X PBS, the fixed samples were lysed in 500 µl cell lysis buffer (Cell lytic buffer, Sigma) and collected nuclei were stored at −80°C as nuclear pellets or nuclear lysates dissolved in nuclei lysis buffer (50 mM Tris-HCl pH8.0, 10-mM EDTA, 1% (wt/vol) SDS and protease inhibitor). The resulting nuclear lysate was sonicated until crosslinked chromatin was sheared to an average length of 0.3~1.0 kb. The supernatant (5 µl) was used as an input control. The remaining lysate was diluted 10-fold with ChIP dilution buffer (16.7 mM Tris–HCl, 167 mM NaCl, 0.01% SDS, 1.1% Triton X-100, 1.2 mM EDTA, and protease inhibitor) and was incubated with Nrf2 antibody, followed by DNA/protein A-agarose magnetic beads. Bound protein-DNA complexes were eluted with a solution containing 0.1 M $NaHCO_3$ and 1% SDS (elution buffer). After the cross-linking was reversed, chromatin fragments were treated with RNase A and proteinase K and DNA was purified with phenol-chloroform-isoamyl alcohol extraction. The DNA sample was then subjected to quantitative real-time PCR using a StepOne Real-Time System (Applied Biosystems, USA) with standard cycling conditions. In the ChIP-qPCR analyses, the values from the immunoprecipitated samples were normalized to that from the input DNA. Primer sequences are provided in Table 1 below.

Immunofluorescence staining and microscopy

The brain coronal tissue (10 µm thickness) sections were washed with PBS and fixed in 4% paraformaldehyde for 20 minutes at 25°C. After blocking the tissue sections for an hour at 25°C with 3% normal goat serum containing 0.1% Triton X-100, the tissue sections were incubated with primary antibodies (3.0 µg/mL) against Annexin-V, Nrf2, pNrf2, HO-1, GLUT-1, 4HNE, ICAM1, LFA-1, PDGF-B, PDGFR-β1, Claudin5, Occludin, Mac-1, and vWF at 4°C overnight (see Table 2 for details of antibodies). To show the cell specificity of the expression of the above-mentioned proteins, in double immunofluorescence, we incubated the tissue sections with anti-GLUT1 or vWF (microvessel or endothelial cell markers) antibodies and co-localized the images. To col-localize Nrf2 or tight junction proteins, we used vWF as microvessel marker to optimize detection sensitivity and specificity for that particular experimental condition. Following three washes with 1X PBS, the tissue sections were incubated in secondary antibody (anti-mouse or anti-rabbit immunoglobulin G) conjugated with AlexaFluor 488 or 594 at a dilution of 1:500 or 4 µg/mL for 1 hour and mounted with 5-10 µL Immuno mount containing DAPI (Invitrogen) on a slide.

Leica fluorescent microscope DMi8 with LAS X software was used to capture the photographs. The stained tissue sections that mounted to slides were selected randomly for quantification, which was carried out in a blind manner by obscuring the slide label. For quantitative analysis, we used a minimum of 6 tissue samples and captured 6 images from a single sample (slide). For consistency, the excitation light brightness, detector sensitivity (gain), and camera exposure duration were maintained constant throughout the capturing of samples or tissue sections. Using ImageJ (NIH) software, the immunostaining intensity was evaluated. To calculate the percentage of positive cells for a given protein, we threshold the images by maintaining lower threshold levels of 80 and an upper threshold level of 200. To rectify uneven illumination in fluorescence images, we maintained a uniformly dark background for every image.

Western Blotting

For western blotting analysis, about 50 mg tissue samples were collected from the frontal cortex area and tissues were lysed with Cell Lytic-M buffer (Thermo Scientific, Rockford, IL) containing a mixture of protease inhibitor (Sigma-Aldrich, St. Louis, MO). In the subsequent procedure, the homogenate was centrifuged at 15,000 rpm for 10 min at 4°C to obtain a clear protein lysate. The total protein was quantified using the bicinchoninic acid (BCA) protein assay kit (Thermo Scientific, Rockford, IL). For immunoblots preparation, 15 µg/lane tissue lysate was used and separated in 4-15% gradient SDS-PAGE gel (Biorad, Hercules, CA). Then the proteins were transferred onto a nitrocellulose membrane, and the membrane was blocked with (5%) dry non-fat milk (Biorad, Hercules, CA) for 1 h. The membrane was incubated with respective primary antibodies (see Table 2 for details of antibodies) overnight at 4°C. Further, the membrane was incubated with horseradish peroxidase-conjugated secondary antibody (1:5000; Fisher Scientific, Rockford, IL) for 1 h at room temperature After the secondary antibody incubation, the membrane was washed 3 times with TBS-tween 20 for 15 min at RT. Protein bands of interest were detected using chemiluminescence western blot detection reagents (Advansta, San Jose, CA) and imaging was processed using the GE ImageQuant LAS-4000 gel documentation system. Furthermore, the optical density was quantified as arbitrary densitometry intensity units using the ImageJ software package (NIH). β-actin was used as a loading control to normalize and quantify proteins of interest.

ELISA

Using specific ELISA kits, the level of Nrf2 (Cat No: LS-F2192-1, LS Bio, Shirley, MA) and ICAM-1 (Cat No: ab203884, Abcam, Waltham, MA), were analyzed in blood plasma and tissue lysate as per manufacturer's instructions.

In vivo BBB permeability assay

The effect of ethanol on BBB permeability was studied in animal models using the sodium fluorescein (NaFl) and Evans Blue (EB) tracer dye mixtures (5 µM each) as previously reported[10,14,15]. After two weeks of NP treatments, the right carotid artery of the animals was surgically exposed and NaFl/EB dye mixture was infused. Two hours after infusion, the animals were perfused transcardially with 150 mL of 1X PBS and 100 mL of 4% PFA, decapitated, and the brains were extracted, weighed, and homogenized in 600 µl of 7.5% (w/v) trichloroacetic acid (TCA). The resulting suspensions were separated into two 300 µl aliquots. For determining the NaFl permeability, one portion of suspension was neutralized with 50 µl of 5 N NaOH and determined through fluorimetry on a microplate reader (excitation 485 nm, emission 535 nm; Promega). To determine EB permeability, the second suspension was centrifuged for 10 minutes at 10,000 rpm and 4°C. The supernatant was then measured using an absorbance spectroscopy (Promega) at 620 nm for EB determination. A standard EB/NaFl solution was serially diluted in 7.5% TCA to create the standard curve.

Transmigration assay *in vivo*

For *in vivo* transmigration assay, we first dissected the femoral bones from euthanized GFP transgenic mice (Strain: C57BL/6-Tg(CAG-EGFP)131Osb/LeySopJ; https://www.jax.org/strain/006567) as we reported recently[16]. Further, under sterile conditions, bone marrow was flushed out repeatedly with 1X HBSS through the cut ends of the bones using a 1 mL syringe. Then, using a 40- mm cell strainer the bone marrow suspension was filtered. The filtrate obtained was collected and centrifuged at 1,800 rpm for 5 min at 4°C. The pellet was collected and suspended in 1 mL of DMEM/F-12 media containing 10% FBS, penicillin, and

streptomycin (100 mg/ml each, Invitrogen) and 0.001% macrophage-colony stimulating factor (MCSF; 500 ml in 500-ml media). Next, the cells were dissociated by trituration (20–25 times) and counted manually with Trypan Blue using a Hemocytometer. Bone marrow-derived cells were differentiated into macrophages in a culture medium (plating 2 x 10^6 cells/T-75 flask) supplemented with MCSF. Cells were fed with media every third day. After 6 days of incubation, the differentiated macrophages were detached and collected using a cell scraper and were suspended in 1 mL HBSS followed by centrifugation at 1800 rpm for 5 min at 4°C. Then the cell pellets were dissociated by repeated trituration and the cells were counted. The GFP^+ bone marrow-derived macrophages (BMM) cells were resuspended in HBSS (2 X10^6 cells) and infused into WT or *Nrf2*$^{-/-}$ ED or CD-fed mice with CP or NP treatments through the tail vein using a 30-G needle Finally, the animal was euthanized 1 h after the cell infusion and brain cortical tissue sections were prepared for observation of GFP^+ macrophages under a fluorescent microscope. To confirm the transmigration of macrophages, we co-immunostained with an anti-Mac-1 antibody (a macrophage marker) and co-localized the images. We validated the role of Nrf2 in the transmigration of leukocytes in *Nrf2*$^{-/-}$ animals and the therapeutic effect of NP was confirmed.

Data analysis

Sample sizes were prospectively derived by conducting power analyses using G*Power (University of Dusseldorf, Germany) based on our previous observations with outcome variations and effect sizes in the mouse model[9-11,17,18]. Our sample sizes were determined with the condition of an 80% chance of detecting a moderate effect size. GraphPad Prism V9 (Sorrento Valley, CA) was used for the statistical analysis of data. The data were tested for normality and equality of variance and analyzed using unpaired t-tests given that our data were collected from independent groups. Interactions between samples/groups will be achieved by three-way ANOVA (since there are three independent variables, liquid diet, genotype, and peptide treatment) followed by Bonferroni post-hoc tests. Data expressed as mean ± SD, and $p < 0.05$ considered for statistical significance. Western blot data were quantified by densitometry analysis using ImageJ software[16,19,20] normalized to β-actin. The immunostaining intensity or the number of positive cells was quantified using the standard method in ImageJ software[16,19,20]. We used a double-blinded study design whereby mice were assigned a unique subject number and then randomized to treatment groups in a predetermined manner by a blinded study coordinator. Blinded investigators performed all data acquisition of outcome measures. Following the final data acquisition, mice were decoded, and final analyses were performed.

TABLE 1: The sequences of RT-qPCR and ChiP-qPCR primers

**RT-qPCR primers:**		
Gene target	FWD primer sequence (5'→ 3')	REV primer sequence (5'→ 3')
GPx1	GATCTCAGCACCATCCAGTT	GGACAGCAGGGTTTCTATGT
GSTm1	CGCTACATCGCAACACCTAT	GGGTAATTCTAGGAAGCGTGAG
HO-1	CTCCCTGTGTTTCCTTTCTCTC	CAGTCGTGGTCAGTCAACAT
NQO1	AGTGCTCGTAGCAGGATTTG	TCTGGTTGTCAGCTGGAATG
SOD	GGTGAACCAGTTGTGTTGTCAGG	ATGAGGTCCTGCACTGGTACAG
Nrf2	GCCTTACTCTCCCAGTGAATAC	CTCCCAAATGGTGCCTAAGA
GAPDH	GGTCGGTGTGAACGGATTT	GTGGATGCAGGGATGATGTT
**ChIP-qPCR primers:**		
Gene target	FWD primer sequence (5'→ 3')	REV primer sequence (5'→ 3')
GPx1	ACA ATA TAA GGG AGC TGT GCG T	CTA GGG CGG GTC TGG TCT A
GSTm1	GGA CAA AGA AAA GGT GGT ACG	TGG GTT AAC TCA CCC AGA ATG
HO-1	TGA AGT TAA AGC CGT TCC GG	AGC GGC TGG AAT GCT GAG T
NQO1	TCT AAG AGC AGA ACG CAG CA	TTC GTG GGA CCT GCC TAC AT

TABLE 2: Details of the antibodies used for this study.

Antibodies	Dilution	Catalog Number	RRID	Vendor
anti-β-actin	WB: 1:1000	MA575739	AB_2545348	ThermoFisher
anti-annexin V	IHC: 1:250	PA5-27872	AB_2545348	ThermoFisher
anti-Nrf2	WB: 1:1000; IHC: 1:250	MAB3925	AB_2263162	R and D
anti-p-Nrf2	WB: 1:1000; IHC: 1:250	PA5-67520	AB_3353597	Novus Biologicals
Anti-Keap1	WB: 1:1000	8047S	Not available	Cell Signaling
anti-GPx1	WB: 1:1000	PA5-30593	AB_2548067	ThermoFisher
anti-GLUT1	IHC: 1:250	ab80024	AB_2190927	Abcam
anti-GSTm1	WB: 1:1000	PA5-22278	AB_11154815	ThermoFisher
anti-HO-1	WB: 1:1000; IHC: 1:250	GTX101147	AB_1950502	Gene Tex
anti-NQO1	WB: 1:1000	ab80588	AB_1603750	Abcam
anti-NOX1	WB: 1:1000	SAB4200097	AB_10620170	Sigma- Aldrich
anti-4HNE	WB: 1:1000; IHC: 1:250	ab46545	AB_722490	Abcam
anti-ICAM-1	WB: 1:1000; IHC: 1:250	MA5407	AB_223596	ThermoFisher
anti-Mac1	WB: 1:1000; IHC: 1:250	nb11089474	AB_1216361	Novus Biologicals
anti-LFA 1	WB: 1:1000; IHC: 1:250	ab186873	Not available	Abcam
anti-occludin	WB: 1:1000; IHC: 1:250	ab31721	AB_881773	Abcam
anti-claudin-5	WB: 1:1000; IHC: 1:250	ab15106	AB_301652	Abcam
anti-JAM-a	WB: 1:1000	Sc53623	AB_784134	Santa Cruz Biotec
anti-vWF	IHC: 1: 250	Ab11713	AB_298501	Abcam
anti-PDGF-B	WB: 1:1000; IHC: 1:250	MAB1739	AB_2299429	ThermoFisher
anti-PDGFR-β1	WB: 1:1000; IHC: 1:250	3169 S	AB_2162497	Cell Signaling
anti-integrin-α6	WB: 1:1000	3750S	Not available	Cell Signaling
anti-integrin-β1	WB: 1:1000	ab183666	AB_3698195	Abcam

IHC: immunohistochemistry; WB: western blotting.

References

1 Dong, Y. *et al.* Metabolomics study of the hepatoprotective effect of Phellinus igniarius in chronic ethanol-induced liver injury mice using UPLC-Q/TOF-MS combined with ingenuity pathway analysis. *Phytomedicine : international journal of phytotherapy and phytopharmacology* **74**, 152697 (2020). https://doi.org/10.1016/j.phymed.2018.09.232

2 Lieber, C. S. & DeCarli, L. M. The feeding of ethanol in liquid diets. *Alcoholism, clinical and experimental research* **10**, 550–553 (1986). https://doi.org/10.1111/j.1530-0277.1986.tb05140.x

3 Muneer, P. M. A. *et al.* Activation of NLRP3 inflammasome by cholesterol crystals in alcohol consumption induces atherosclerotic lesions. *Brain, behavior, and immunity* **62**, 291–305 (2017). https://doi.org/10.1016/j.bbi.2017.02.014

4 Muneer, P. M. A., Alikunju, S., Szlachetka, A. M. & Haorah, J. Inhibitory effects of alcohol on glucose transport across the blood-brain barrier leads to neurodegeneration: preventive role of acetyl-L: -carnitine. *Psychopharmacology* **214**, 707–718 (2011). https://doi.org/10.1007/s00213-010-2076-4

5 Muneer, P. M. A., Alikunju, S., Szlachetka, A. M. & Haorah, J. The mechanisms of cerebral vascular dysfunction and neuroinflammation by MMP-mediated degradation of VEGFR-2 in alcohol ingestion. *Arteriosclerosis, thrombosis, and vascular biology* **32**, 1167–1177 (2012). https://doi.org/10.1161/ATVBAHA.112.247668

6 Alikunju, S., Muneer, P. M. A., Zhang, Y., Szlachetka, A. M. & Haorah, J. The inflammatory footprints of alcohol-induced oxidative damage in neurovascular components. *Brain, behavior, and immunity* **25 Suppl 1**, S129–136 (2011). https://doi.org/10.1016/j.bbi.2011.01.007

7 Dalbeyler, K. *Tissue Dissection of Mouse Brain Regions: Prefrontal Cortex, Motor Cortex, and Somatosensory Cortex [PhD Thesis]. Heidelberg University.* Ph.D thesis, Heidelberg University., (2021).

8 Muneer, P. M. A. *et al.* Impairment of Thiamine Transport at the GUT-BBB-AXIS Contributes to Wernicke's Encephalopathy. *Molecular neurobiology* **55**, 5937–5950 (2018). https://doi.org/10.1007/s12035-017-0811-0

9 Bhowmick, S., D'Mello, V. & Muneer, P. M. A. Synergistic Inhibition of ERK1/2 and JNK, Not p38, Phosphorylation Ameliorates Neuronal Damages After Traumatic Brain Injury. *Molecular neurobiology* **DOI:10.1007/s12035-018-1132-7.** (2018). https://doi.org/10.1007/s12035-018-1132-7

10 Bhowmick, S., D'Mello, V., Caruso, D., Wallerstein, A. & Muneer, P. M. A. Impairment of pericyte-endothelium crosstalk leads to blood-brain barrier dysfunction following traumatic brain injury. *Experimental neurology* **317**, 260–270 (2019). https://doi.org/10.1016/j.expneurol.2019.03.014

11 Bhowmick, S., Dmello, V., Caruso, D. & Muneer, P. M. A. Traumatic brain injury-induced down regulation of Nrf2 activates inflammatory response and apoptotic cell death. . *Journal of Molecular Medicine* **97(12):1627-1641. doi: 10.1007/s00109-019-01851-4.** (2019).

12 Porte, B. *et al.* Proteomic and transcriptomic study of brain microvessels in neonatal and adult mice. *PloS one* **12**, e0171048 (2017). https://doi.org/10.1371/journal.pone.0171048

13 Lee, Y. K., Uchida, H., Smith, H., Ito, A. & Sanchez, T. The isolation and molecular characterization of cerebral microvessels. *Nature protocols* **14**, 3059–3081 (2019). https://doi.org/10.1038/s41596-019-0212-0

14 Hawkins, B. T. & Egleton, R. D. Fluorescence imaging of blood-brain barrier disruption. *Journal of neuroscience methods* **151**, 262–267 (2006). https://doi.org/10.1016/j.jneumeth.2005.08.006

15 Muneer, P. M. A. *et al.* Induction of oxidative and nitrosative damage leads to cerebrovascular inflammation in an animal model of mild traumatic brain injury induced by primary blast. *Free radical biology & medicine* **60**, 282–291 (2013). https://doi.org/10.1016/j.freeradbiomed.2013.02.029
16 Saikia, B. B. *et al.* ICAM-1 deletion using CRISPR/Cas9 protects the brain from traumatic brain injury-induced inflammatory leukocyte adhesion and transmigration cascades by attenuating the paxillin/FAK-dependent Rho GTPase pathway. *The Journal of neuroscience : the official journal of the Society for Neuroscience* (2024). https://doi.org/10.1523/JNEUROSCI.1742-23.2024
17 Patel, R. K., Prasad, N., Kuwar, R., Haldar, D. & Muneer, P. M. A. Transforming growth factor-beta 1 signaling regulates neuroinflammation and apoptosis in mild traumatic brain injury. *Brain, behavior, and immunity* (2017). https://doi.org/10.1016/j.bbi.2017.04.012
18 Bhowmick, S. *et al.* Intercellular adhesion molecule-1-induced post-traumatic brain injury neuropathology in the prefrontal cortex and hippocampus leads to sensorimotor function deficits and psychological stress. *eNeuro* (2021). https://doi.org/10.1523/ENEURO.0242-21.2021
19 Bhowmick, S., Alikunju, S. & Muneer, P. M. A. NADPH oxidase-induced activation of transforming growth factor-beta-1 causes neuropathy by suppressing antioxidant signaling pathways in alcohol use disorder. *Neuropharmacology* **213**, 109136 (2022). https://doi.org/10.1016/j.neuropharm.2022.109136
20 Muneer, P. M. A., Saikia, B. B. & Bhowmick, S. Synergistic effect of mild traumatic brain injury and alcohol aggravates neuroinflammation, amyloidogenesis, tau pathology, neurodegeneration, and blood-brain barrier alterations: Impact on psychological stress. *Experimental neurology* **358**, 114222 (2022). https://doi.org/10.1016/j.expneurol.2022.114222
