# Supplementary material for: Nrf2 activator peptide protects the brain from cerebral vascular dysfunction in alcohol ingestion
Source: JCI Insight. 2026 Feb 17;11(6):e188004. doi: 10.1172/jci.insight.188004 (PMC13043087; doi:10.1172/jci.insight.188004)
Supplement: Unedited blot and gel images [file jciinsight-11-188004-s024.pdf]

Uncropped western blotting images.

Figure 2A

Nrf2

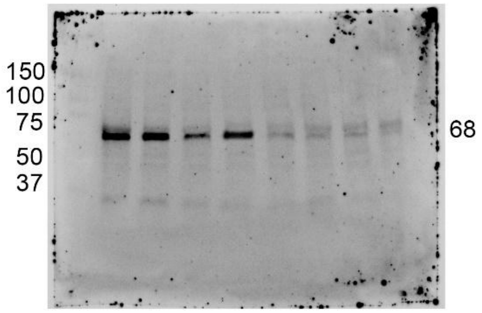

p-Nrf2

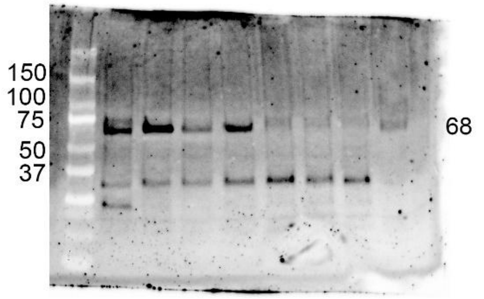

β-actin

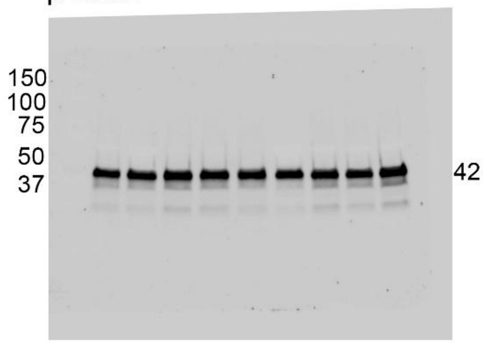

Figure 2E

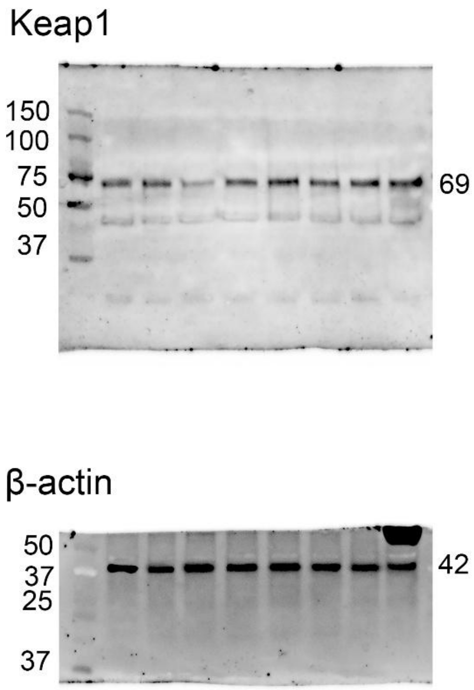

Figure 2H

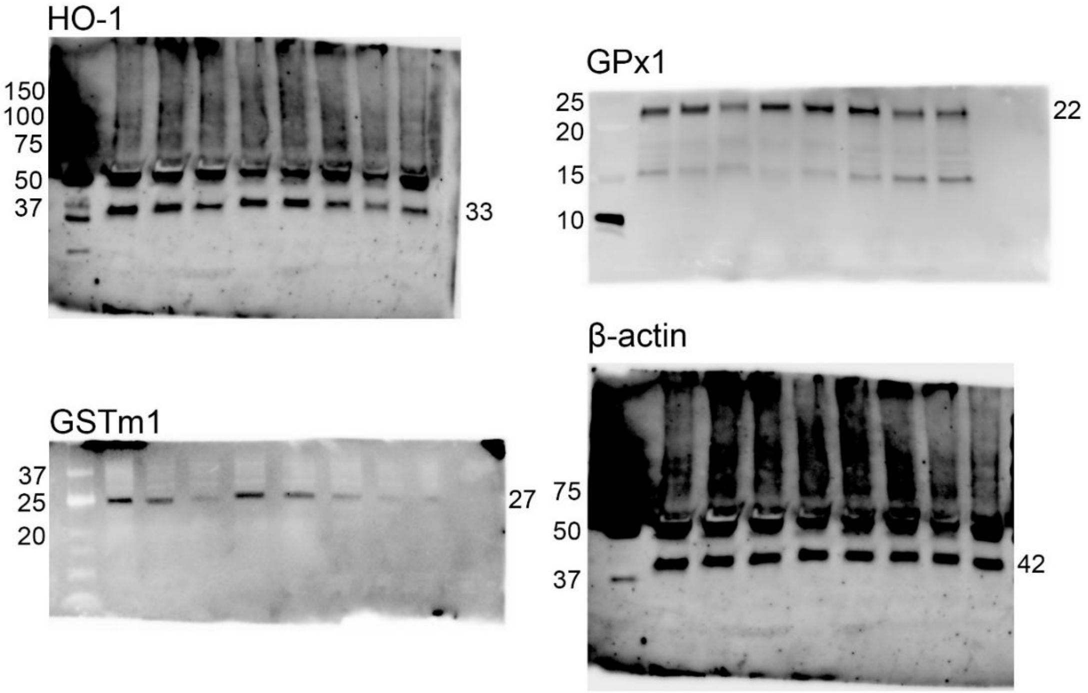

Figure 4C

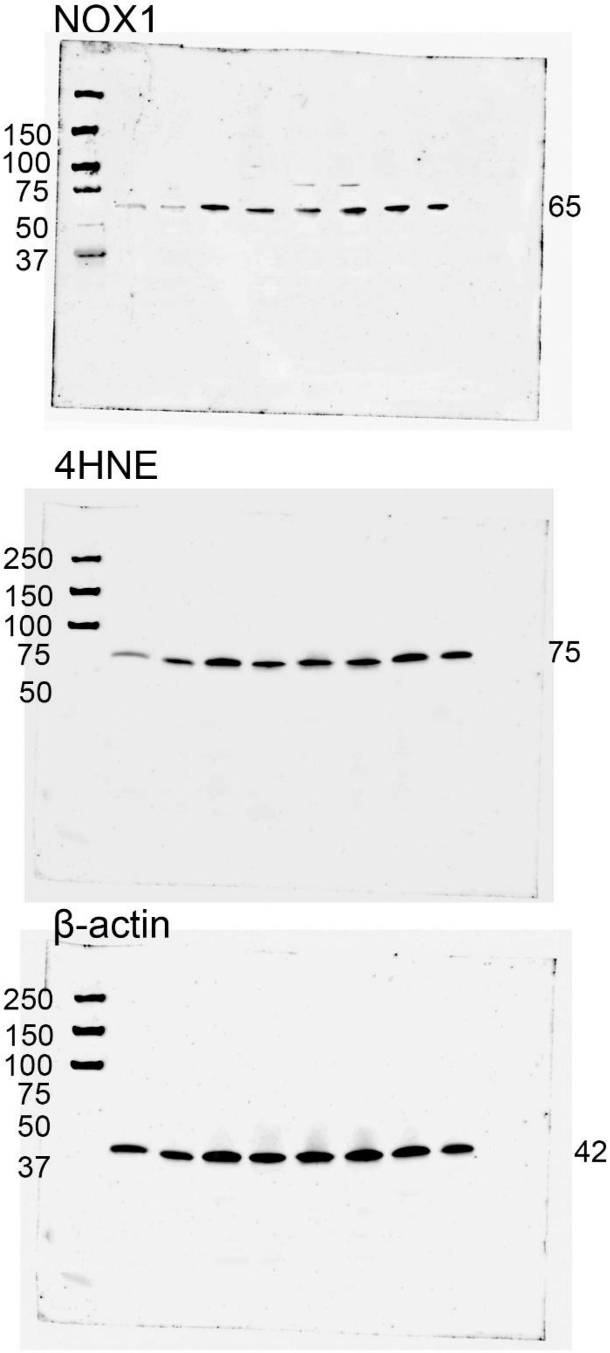
NOX1
150
100
75
50
37
65
4HNE
250
150
100
75
50
75
β-actin
250
150
100
75
50
37
42

Figure 5A

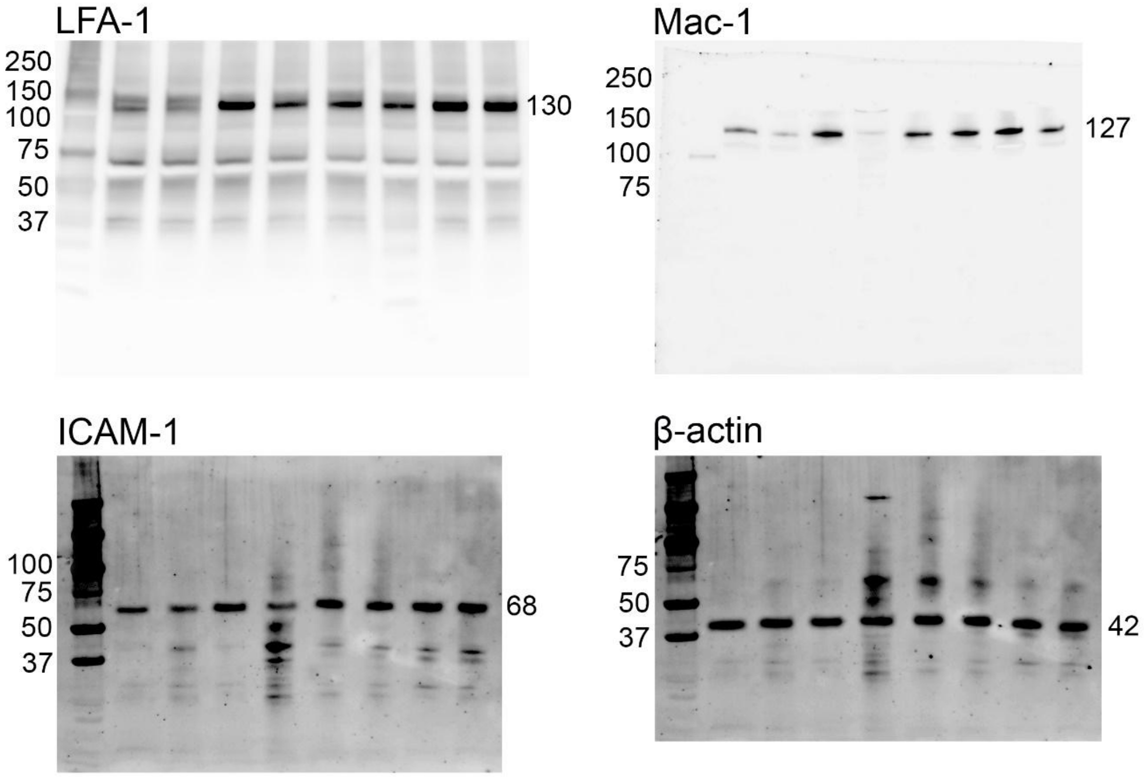

LFA-1
250
150
100
75
50
37
130
Mac-1
250
150
100
75
127
ICAM-1
100
75
50
37
68
β-actin
75
50
37
42

Figure 6D

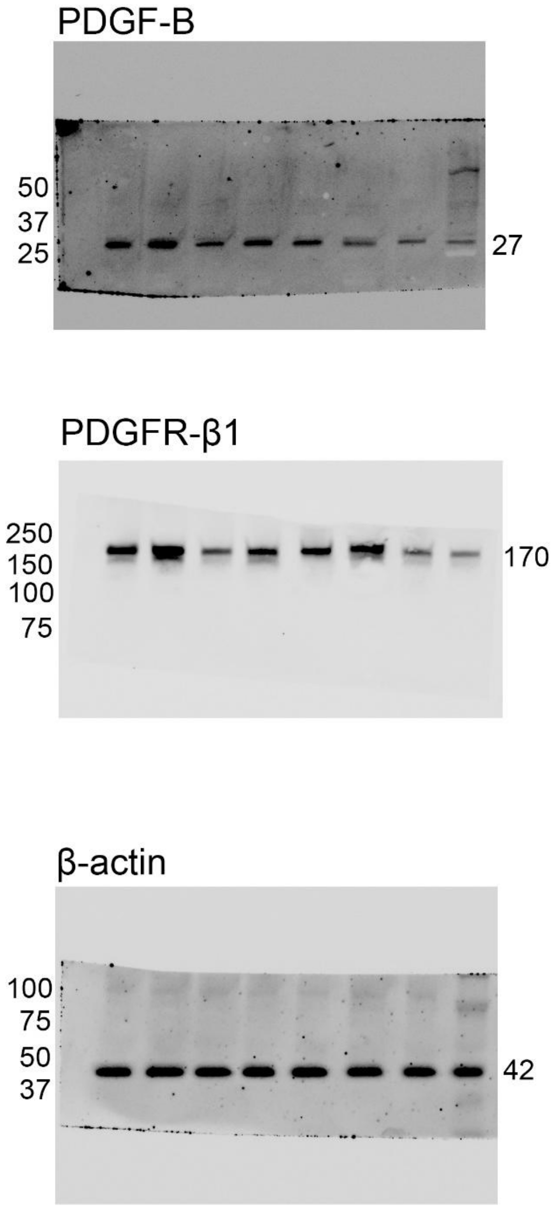

Figure 6E

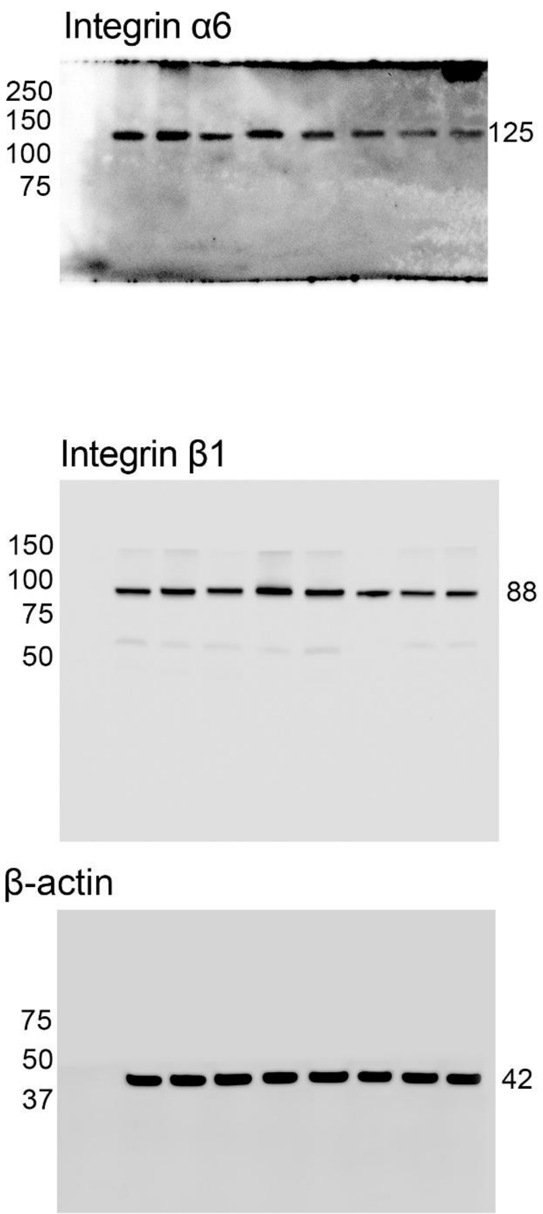

Figure 7E

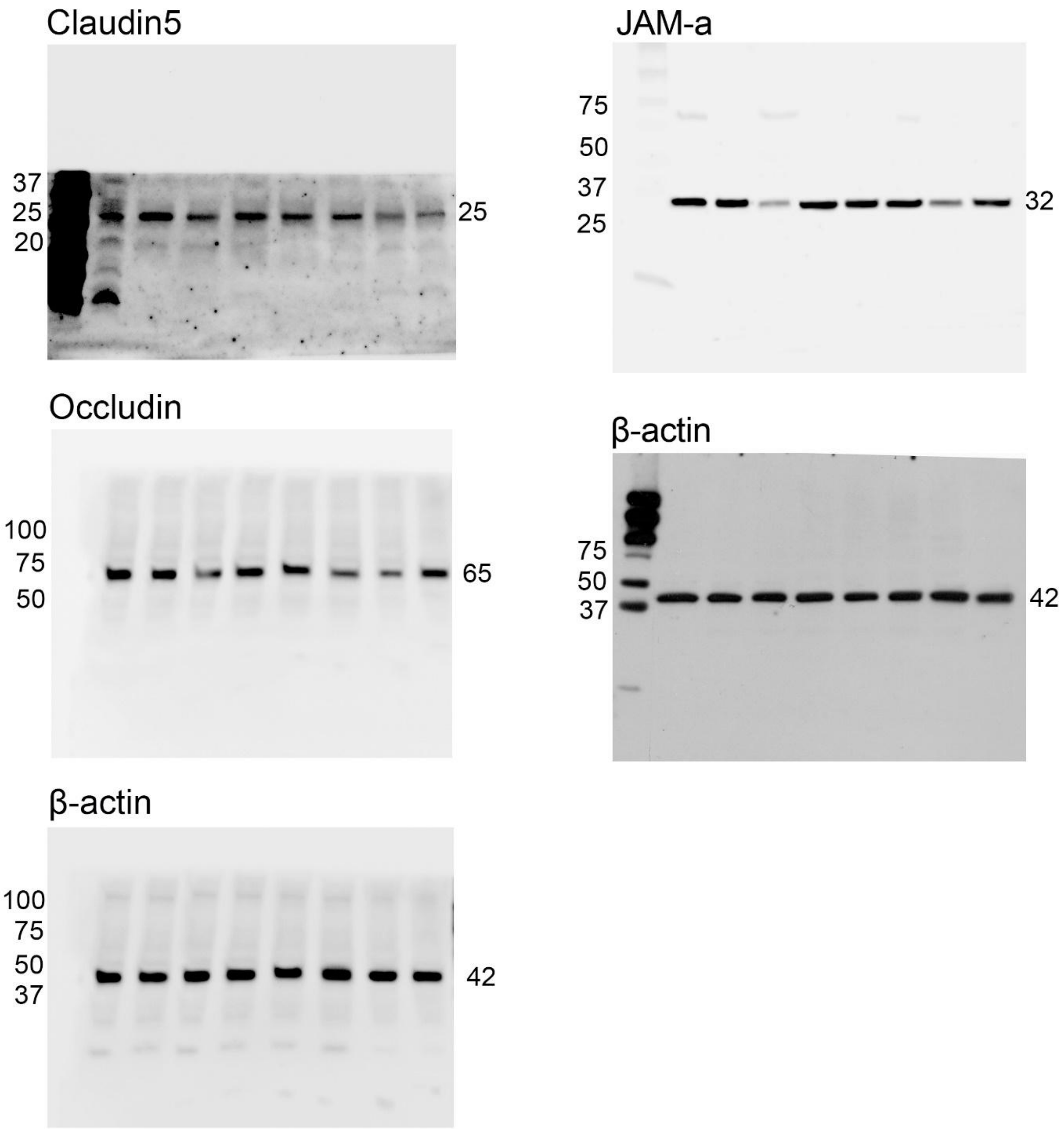

Claudin5
37
25
20
25
JAM-a
75
50
37
25
32
Occludin
100
75
50
65
β-actin
75
50
37
42
β-actin
100
75
50
37
42
